# Objective oculomotor, vestibular, reaction time, and cognitive signatures of vestibular migraine

**DOI:** 10.3389/fneur.2026.1789811

**Published:** 2026-05-20

**Authors:** Claire E. J. Ceriani, Alexandr Braverman, Alexander Kiderman

**Affiliations:** 1 Department of Neurology, Thomas Jefferson University, Philadelphia, PA, United States; 2 Spryson America, Inc., Pittsburgh, PA, United States

**Keywords:** cognitive function, dizziness, eye tracking, headache, oculomotor function, reaction time, vestibular function, vestibular migraine

## Abstract

**Objective:**

To evaluate oculomotor, vestibular, reaction time, and cognitive (OVRT-C) function in patients with vestibular migraine (VM) using objective eye-tracking–based metrics and to identify patterns of dysfunction relative to healthy controls.

**Background:**

Vestibular migraine is a common yet underdiagnosed cause of vertigo. Diagnosis remains primarily clinical, because objective neurologic and vestibular findings are often absent or inconsistent. Quantitative methods capable of capturing the multisystem manifestations of VM may improve objective characterization of the disorder.

**Methods:**

Participants with a clinical diagnosis of vestibular migraine were recruited from a tertiary headache center and assessed using a battery of OVRT-C tests administered with a portable eye-tracking system (Spryson Dx-100; *n* = 52). The test battery assessed gaze stability, saccades, antisaccades, smooth pursuit, vergence, optokinetic responses, and visual and auditory reaction times. Participants also underwent bedside neurologic and vestibular examinations and completed the Dizziness Handicap Inventory (DHI). OVRT-C metrics were compared with normative data from a database of 300 healthy adults. Univariate and stepwise multivariate logistic regression models were used to identify metrics that differentiated VM patients from controls.

**Results:**

A substantial proportion of VM patients aged 18 to 45 demonstrated abnormal OVRT-C performance compared with normative data, most prominently in horizontal and vertical saccades (54.3 and 51.4%, *p* < 0.0001), vertical smooth pursuit (62.9%, *p* < 0.0001), optokinetic responses (43.8%, *p* < 0.0001), and gaze stability (65.7%). Several OVRT-C metrics showed strong discriminative ability in logistic regression analyses when considering a single OVRT-C metric is considered (adjusted for participant age and gender). A multiple logistic regression model identified six OVRT-C metrics as significant indicators of VM and demonstrated excellent classification performance (AUC = 0.996), with estimated specificity of 95.9% and sensitivity of 99.7% for probability cutoff of 0.5. Moderate but statistically significant correlations were observed between OVRT-C metrics, bedside neurologic and vestibular findings, and DHI domain scores.

**Conclusion:**

Objective OVRT-C testing reveals quantifiable abnormalities in oculomotor, vestibular, and cognitive dysfunction in patients with vestibular migraine. These findings support the feasibility of eye-tracking–based multimodal assessments as complementary tools for characterizing vestibular migraine and warrant further validation in larger and longitudinal cohorts.

## 1 Introduction

Dizziness is the second most reported symptom in migraine after headache pain (1). In 1999, Dieterich and Brandt introduced the term vestibular migraine (VM) to describe episodic vertigo occurring in association with migraine (2). VM is now recognized as the most common neurologic cause of vertigo (3), with an estimated prevalence of 0.98–2.7% in the adult population (4, 5). Despite its prevalence, VM remains underdiagnosed likely due to the wide range of clinical presentations and the fact that many patients report dizziness or vertigo as more disabling than headache pain (6, 7). More than half of patients demonstrate a normal neuro-otologic examination during the interictal period, necessitating reliance on clinical history for diagnosis (6).

In 2012, the International Headache Society and the International Bárány Society established diagnostic criteria for vestibular migraine, which were subsequently incorporated into the third edition of the International Classification of Headache Disorders (ICHD-3) in 2018 (Box 1) (8, 9). Although these criteria have improved diagnostic consistency, no single vestibular or oculomotor abnormality has been identified as pathognomonic for VM.

Box 1 **Diagnostic criteria for vestibular migraine [taken from Appendix A1.6.6 in Headache Classification Committee of the International Headache Society (9)]**

- At least five episodes fulfilling criteria C and D
- A current or past history of migraine without aura or migraine with aura
- Vestibular symptoms of moderate or severe intensity, lasting between 5 min and 72 h
- At least half of episodes are associated with at least one of the following three migrainous features:

1. Headache with at least two of the following four characteristics:

a) Unilateral location
b) Pulsating quality
c) Moderate or severe intensity
d) Aggravation by routine physical activity

2 Photophobia and phonophobia
3 Visual aura

E Not better accounted for by another ICHD-3 diagnosis or by another vestibular disorder

Vestibular symptoms, as defined by the Bárány Society’s Classification of Vestibular Symptoms and qualifying for a diagnosis of *Vestibular migraine*, include:

a) Spontaneous vertigo:

a Internal vertigo (a false sensation of self-motion)
b External vertigo (a false sensation that the visual surround is spinning or flowing)

b) Positional vertigo, occurring after a change in head position
c) Visually-induced vertigo, triggered by a complex or large moving visual stimulus
d) Head motion-induced vertigo, occurring during head motion
e) Head motion-induced dizziness with nausea (dizziness is characterized by a sensation of disturbed spatial orientation; other forms of dizziness are currently not included in the classification of vestibular migraine)

Nevertheless, abnormalities on vestibular and oculomotor testing have been reported in VM patients (10–13). For example, in patients presenting with dizziness or vertigo, individuals with migraine or tension-type headache have demonstrated greater deviations on subjective visual vertical (SVV) testing compared with those without headache (14). Migraine patients also exhibit mild postural instability on static posturography, which worsens during migraine attacks (15, 16). Reduced vestibular-evoked myogenic potential (VEMP) amplitudes have been observed in VM patients (17), with one study reporting absent unilateral or bilateral VEMP responses in 44% of VM patients and 25% of patients with migraine without vestibular symptoms (18). Collectively, these findings suggest that vestibular dysfunction is an integral component of VM pathophysiology and may also be present subclinically in migraine without overt vestibular symptoms (7).

An emerging area of investigation involves the assessment of oculomotor, vestibular, reaction time, and cognitive (OVRT-C) responses to visual and vestibular stimuli. Patients with VM frequently report visually induced dizziness, spatial disorientation, cognitive slowing, and “brain fog” (6), suggesting multisystem neural involvement beyond vertigo alone. The aim of this study was to evaluate a comprehensive OVRT-C testing battery to objectively characterize vestibular and neurologic dysfunction in patients with vestibular migraine and to determine whether specific patterns of impairment may aid in diagnosis.

## 2 Materials and methods

### 2.1 Study design and ethics approval

This prospective study was approved by the Institutional Review Board of Thomas Jefferson University. All participants provided written informed consent prior to enrollment.

### 2.2 Participants

**Figure 1:**
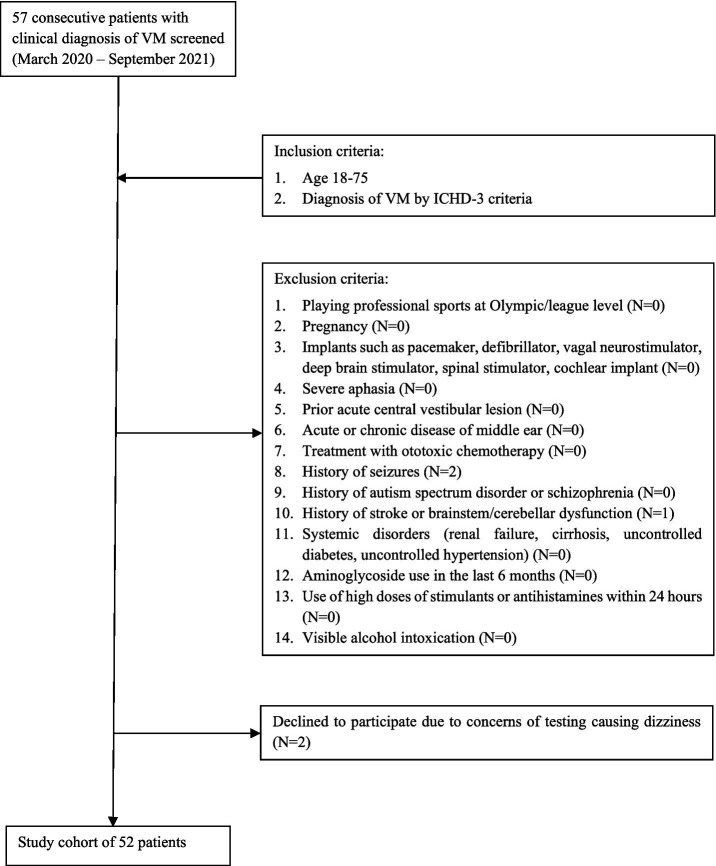
Study recruitment.

Adults aged 18–75 years were recruited from a tertiary headache center in Philadelphia, PA. Participants were eligible for inclusion if they had a clinical diagnosis of vestibular migraine confirmed by a headache medicine specialist according to ICHD-3 criteria. Exclusion criteria included neurologic, vestibular, or other medical conditions that could affect test performance or limit the ability to follow instructions (see Figure 1 for full exclusion criteria).

A total of 57 patients were screened. Two were excluded due to a history of seizures, one due to cerebellar atrophy, and two declined participations because of concern that testing might exacerbate symptoms. Fifty-two participants aged 18–70 years (mean age 40.6 ± 14.1 years) were enrolled; 42 participants (80.8%) were women (Table 1). Tests were administered in a standardized sequence. Horizontal and vertical tests were interleaved to mitigate fatigue or adaptation effects. Pilot analyses indicated no systematic impact of test order on key OVRT-C outcomes. All enrolled participants completed OVRT-C testing.

**Table 1:** Participant demographics.

Testing sites	*N*	Age				Gender			
		Min	Max	Mean	SD	F	*%*	M	*%*
Vestibular migraine data									
Jefferson Headache Center, Thomas Jefferson University Hospitals, Philadelphia, PA	52	18	70	40.6	14.1	42	80.8	10	19.2
Normative data^§^									
Naval Med. Center San Diego, San Diego, CA	48	18	45	28.0	6.2	14	29.2	34	70.8
Madigan Army Med. Center, Fort Lewis, W-ton	252	18	45	27.3	6.3	81	32.1	171	67.9
Total	300	18	45	27.4	6.3	95	31.7	205	68.3

^§^Normative data participants were tested at two sites and are representative of a healthy population.

Normative control data were obtained from previously collected and published datasets (19, 20). These FDA-approved normative data included 300 healthy adults aged 18–45 years (mean age 27.4 ± 6.3 years), of whom 31.7% were women, recruited from two testing sites (Table 1).

### 2.3 Subject evaluation

Study visits were conducted between March 2020 and September 2021 by a headache medicine specialist. During a single study visit, participants underwent confirmation of vestibular migraine diagnosis using an investigator-administered questionnaire based on ICHD-3 criteria, a bedside neurologic and vestibular examination, and OVRT-C testing. All but one participant completed the Dizziness Handicap Inventory (DHI).

Vestibular symptoms were categorized according to symptom types recognized by ICHD-3 as qualifying for a diagnosis of vestibular migraine (Box 1) (9). Participants were permitted to report more than one symptom category. All participants tolerated OVRT-C testing without adverse events.

### 2.4 OVRT-C test battery

Participants completed a battery of OVRT-C tests assessing gaze stability, saccades, antisaccades, smooth pursuit, vergence, optokinetic responses, and visual and auditory reaction times (see Supplementary Table S1 to this paper). Each test was designed to quantify performance under controlled visual and vestibular stimuli, providing objective measures of oculomotor control, visual–vestibular integration, and cognitive response. Visual stimuli were presented at high contrast against a neutral background. Individual tests ranged from 7 to 90 s in duration, with a total testing time of approximately 15 min. Participants were seated adjacent to the test administrator, and head stabilization was not used.

### 2.5 Hardware and software

OVRT-C testing was performed using an FDA-cleared portable video-nystagmography (VNG) eye-tracking system (Dx-100; Spryson America, Inc., Pittsburgh, PA, United States). The system consists of lightweight goggles with a soft gasket to ensure a light-tight fit. Visual stimuli were presented using the Dx-100 HMD in a head-free environment. Participants were instructed to minimize head movement during testing. The HMD and high-resolution binocular infrared cameras are rigidly mounted as a single unit, ensuring that any head movement results in synchronous movement of both the display and cameras. Integrated motion sensors continuously monitor the orientation of the Dx-100. Comparison testing with conventional VNG displays confirmed that oculomotor measurements obtained with the HMD were highly reliable relative to the standard VNG system. The HMD provides a 60° field of view with a resolution of 2,560 × 1,440 pixels and supports three-dimensional image perception.

Eye movements were recorded binocularly using integrated infrared cameras at a sampling rate of 100 frames per second with near-frontal infrared illumination (940 nm). The system tracks eye movements within ranges of ±30° horizontally, ±30° vertically, and ±10° torsionally, with an angular resolution of <0.1°. Reaction time tasks were performed using the same VNG system and synchronized via VEST^™^ software.

*Visual reaction time (VRT):* Participants viewed a high-contrast dot appearing randomly at the center of the visual field and responded using a handheld button with their dominant hand.
*Auditory reaction time (ART):* Participants responded to brief auditory tones delivered through headphones using the same handheld button.
*Saccade–reaction time (SRT):* Participants made a saccade toward a visual target while simultaneously pressing a left or right button corresponding to the saccade direction, capturing combined sensory, motor, and cognitive performance.

All OVRT-C metrics were automatically calculated by VEST™ software, which uses validated algorithms to detect saccades, smooth pursuit, optokinetic responses, and reaction times. A trained analyst reviewed all data for quality control, excluding trials affected by blinks, calibration errors, or goggle slippage. The random saccade test presented targets with randomized amplitudes and interstimulus intervals.

Tests were administered in a standardized fixed sequence, with horizontal and vertical tasks interleaved to minimize fatigue or adaptation effects.

### 2.6 Data analysis

Eye movement and reaction time data were continuously recorded during each test. A trained data analyst reviewed all data to confirm test completion and verify signal validity. Any technical issues, such as incomplete trials or poor eye-tracking signal, were flagged for review.

Validated data were analyzed using VEST™ software, which automatically calculates OVRT-C performance metrics corresponding to the measures listed in Supplementary Table S1. For each completed and quality-verified test, metrics were extracted for further statistical analysis. If a test’s data quality was insufficient for reliable analysis, that test result was excluded for the participant. Exclusions were rare and are explicitly noted in the Results where applicable. This approach ensured that all analyzed metrics reflect accurate and valid OVRT-C performance.

### 2.7 Statistical analysis

The primary objective of this study was to evaluate OVRT-C metrics in patients with vestibular migraine (VM) and to identify patterns of dysfunction that differentiate VM patients from healthy controls. Several complementary statistical approaches were employed.

#### 2.7.1 Abnormal rate analysis

For each OVRT-C metric, individual measurements from VM participants (*n* = 52) were compared with the normative reference interval derived from a healthy control database (19, 20). Normative ranges were defined as the 95% reference interval (95% RI). The proportion of VM participants whose values fell outside the 95% RI was calculated for each metric and reported as the observed abnormal rate.

**Figure 2:**
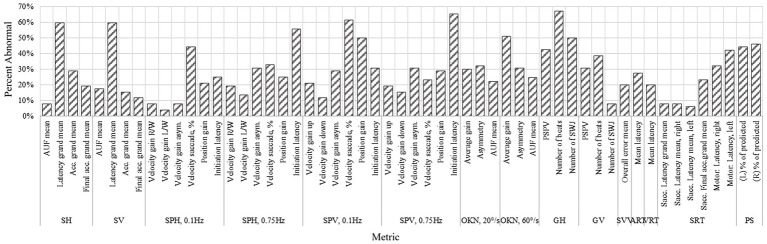
Comparison of test results of patients aged 18–45 (VM 1), patients aged 46–70 (VM 2), and healthy participants (H) with box plots.

In a normative population, 5% of values are expected to fall outside the 95% RI. Observed abnormal rates in VM patients were interpreted relative to this expected value. A one-proportion Z-test was used to assess whether the observed abnormal rate differed significantly from 5%. The test statistic was calculated as:


$$Z=\frac{|\hat{p}-P|-c}{\sqrt{P(1-P)/N}}$$

where $\hat{p}$ is the observed rate of VM subjects whose test value is outside the 95% RI, *P* = 0.05 is an expected normative rate, N is the total number of VM participants, and c=1/(2N) is a correction for continuity. The corresponding *p*-value was calculated under a two-tail hypothesis. The difference was significant when *p* ≤ 0.05. The FDA-approved “95% RI limits” are computed using a normative database that included both male and female volunteers, aged 18–45, without neurological or vestibular disorders. Since the normative database is based on healthy individuals aged 18 to 45, in addition to 52 VM patients (aged 18 to 70), abnormal rates are also reported separately for 35 (67.3% of 52) patients aged 18 to 45 (Figure 2).

#### 2.7.2 Mean difference analysis

For each metric, to determine statistical significance, a Student’s t-test is used to assess whether a difference exists between the mean values of two independent cohorts, that is, between VM patients (*n* = 52) and healthy controls (*n* = 300). The test was performed under the assumption of unequal variances (Behrens–Fisher problem), with Satterthwaite’s approximation for degrees of freedom. Statistical analyses were conducted using the TTEST2 function in MATLAB (The MathWorks, Inc., United States; version R2015b). Two-tailed *p*-values were reported, with significance defined as *p* ≤ 0.05. For certain metrics measured on a signed scale where directionality lacks clinical meaning (e.g., asymmetry or error metrics), mean comparisons were considered less informative. In such cases, abnormal rate analyses were emphasized, and absolute values were not used for statistical testing.

#### 2.7.3 Logistic regression analysis

Logistic regression model (with logit link) is used both to evaluate identify OVRT-C metrics to that can explain the difference (i.e., discriminate) between VM patients and healthy controls when controlling for age (in years) and gender (males coded as 1 and females as 0), and to express the predictive power. The outcomes are defined in terms of predicting VM (coded 1) versus healthy controls (coded 0). The model is fitted separately for each OVRT-C metric using the FITGLM function in MATLAB with maximum likelihood estimation. The regression coefficients and associated *p*-values are computed, and the coefficients are considered when *p* ≤ 0.05. To assess diagnostic (discriminative) ability, the area under the receiver operating characteristic curve (AUC) and Somers’ D statistic (= 2 × AUC − 1) are calculated. Since the AUC for the binary case ranges from 0.5 to 1, using Somers’ D (which ranges from 0 to 1), which measures an ordinal association (rank correlation of observed binary responses and predicted probabilities), is more convenient for interpreting the results.

A multiple logistic regression model was subsequently constructed using a standard stepwise selection procedure to identify the combination of OVRT-C metrics that best distinguished VM patients from controls. Model performance was evaluated using AUC, sensitivity and specificity with a cutoff value of 0.5, and the corresponding 95% confidence intervals. Of note, *sensitivity* is the proportion of event responses that were predicted as events (i.e., VM), and *specificity* is the proportion of non-event responses that were predicted as non-events (i.e., healthy controls). To assess potential overfitting, leave-one-out cross-validation (LOOCV) was performed. Confidence intervals for both the full dataset and LOOCV estimates were obtained using bootstrap resampling with 2,000 replicates.

#### 2.7.4 Correlation analyses

Relationships between OVRT-C metrics and vestibular migraine symptoms were examined using Spearman’s rank correlation coefficient (rho) (Table 2; Supplementary Table S4). Correlations were computed using the CORR function in MATLAB. Two-tailed *p*-values were reported. Given the relatively small and homogeneous sample (*n* = 52), correlations with 0.05 < *p* ≤ 0.10 were interpreted as a tendency toward association.

**Table 2:** Vestibular and related symptoms and abnormal test results in 52 VM subjects.

Symptoms and tests	Frequency	%
Vestibular symptoms		
Spontaneous internal vertigo	41	78.8
Visually induced vertigo	39	75.0
Head motion-induced vertigo	33	63.5
Spontaneous external vertigo	31	59.6
Positional vertigo	12	23.1
Head motion-induced dizziness with nausea	11	21.1
Related symptoms		
Tinnitus	31	59.6
Visual lag	19	36.5
Oscillopsia	19	36.5
Ear pain	16	30.8
Vomiting	15	28.8
Visual snow	10	19.2
Decreased hearing	9	17.3
Alice in wonderland syndrome	3	5.8
Abnormal test results		
Sharpened Romberg	26	50.0
Tandem gait	14	26.9
Weber	4	7.7
Romberg	2	3.8
Fukuda stepping test	2	3.8
Rinne	1	1.9

Associations between OVRT-C metrics and Dizziness Handicap Inventory (DHI) scores were assessed in 51 VM participants (one participant did not complete the DHI). The DHI total score (range 0–100) and domain scores (physical, emotional, functional) were analyzed separately.

## 3 Results

OVRT-C metrics that differentiated VM patients from healthy controls were identified using abnormal rate analysis, mean comparisons, and logistic regression modeling. Abnormal rates and mean differences are summarized in Table 3 (for extended table with a broader list of metrics, the reader is referred to Supplementary Table S2 to this paper). The logistic regression results are presented in Table 4. Pilot analyses indicated no systematic impact of test order on key OVRT-C outcomes, and the findings show that the OVRT-C metric explain the difference between patients and healthy controls, beyond the effect of age and gender. Due to space limitations, the results related to age and gender (i.e., regression coefficients and corresponding significance levels) are not included.

**Table 3:** Abnormal rates among VM participants and student’s t-test to evaluate the mean difference between VM and healthy participants.

Tests	Metrics	t-test (mean difference)							Abnormal rate							
		VM			Healthy			*p*	95% RI limits		VM (All)			VM (Age ≤ 45)		
		N	Mean	SD	N	Mean	SD	(*Mean* *Diff*)	Lower	Upper	*N* Abn	% Abn	*p*	*N* Abn	% Abn	*p*
Saccade— Random, Horizontal (SH)	Latency grand mean	52	0.24	0.04	300	0.18	0.02	0.000	n/a	0.22	31	59.6%	0.000	19	54.3%	0.000
	Accuracy grand mean	52	89.0	10.4	300	92.5	5.6	0.020	81	103	15	28.8%	0.000	11	31.4%	0.000
	Final accuracy grand mean	52	96.0	6.1	300	96.3	3.6	0.711	89	104	10	19.2%	0.000	9	25.7%	0.000
	Area under fit (AUF) mean	52	10,167	1,312	300	10,357	1,026	0.325	8,239	n/a	4	7.7%	0.567	3	8.6%	0.561
Saccade— Random, Vertical (SV)	Latency grand mean	52	0.25	0.03	300	0.19	0.02	0.000	n/a	0.23	31	59.6%	0.000	18	51.4%	0.000
	Accuracy grand mean	52	88.9	12.6	300	92.7	9.5	0.042	75	109	8	15.4%	0.002	4	11.4%	0.175
	Final accuracy grand mean	52	95.2	9.0	300	94.2	7.4	0.461	79	107	6	11.5%	0.065	5	14.3%	0.033
	Area under fit (AUF) mean	52	8,832	1,472	300	9,684	1,339	0.000	7,630	n/a	9	17.3%	0.000	6	17.1%	0.004
Smooth Pursuit— Horizontal (SPH) 0.1 Hz	Velocity gain rightward	52	0.93	0.11	300	0.95	0.08	0.122	0.78	1.07	4	7.7%	0.567	4	11.4%	0.175
	Velocity saccade, %	52	33.6	14.3	300	18.0	10.2	0.000	n/a	35	23	44.2%	0.000	15	42.9%	0.000
	Initiation latency	52	284	81	—	—	—	—	n/a	335	13	25.0%	0.000	8	22.9%	0.000
Smooth Pursuit— Horizontal (SPH) 0.75 Hz	Velocity gain rightward	52	0.87	0.21	300	0.95	0.10	0.010	0.62	1.08	10	19.2%	0.000	7	20.0%	0.000
	Velocity saccade, %	52	30.9	14.2	300	15.8	11.5	0.000	n/a	37	17	32.7%	0.000	8	22.9%	0.000
	Initiation latency	52	268	60	—	—	—	—	n/a	252	29	55.8%	0.000	19	54.3%	0.000
Smooth Pursuit— Vertical (SPV) 0.1 Hz	Velocity gain up	52	0.88	0.17	300	0.90	0.11	0.515	0.69	1.07	11	21.2%	0.000	6	17.1%	0.004
	Velocity saccade, %	52	39.40	17.76	300	14.1	8.5	0.000	n/a	32	32	61.5%	0.000	22	62.9%	0.000
	Initiation latency	52	282.3	90.3	—	—	—	—	n/a	311	16	30.8%	0.000	14	40.0%	0.000
Smooth Pursuit— Vertical (SPV) 0.75 Hz	Velocity gain up	52	0.75	0.26	300	0.81	0.18	0.127	0.42	1.09	10	19.2%	0.000	7	20.0%	0.000
	Velocity saccade, %	52	38.9	16.1	300	26.9	14.5	0.000	n/a	52	12	23.1%	0.000	7	20.0%	0.000
	Initiation latency	52	277	81	—	—	—	—	n/a	230	34	65.4%	0.000	24	68.6%	0.000
Optokinetic (OKN) 20°/sec	Average gain	50	0.77	0.14	300	0.86	0.08	0.000	0.66	0.97	15	30.0%	0.000	12	36.4%	0.000
	Area under fit (AUF) mean	50	7,314	1,636	300	8,274	1,447	0.000	5,735	n/a	11	22.0%	0.000	6	18.2%	0.002
Optokinetic (OKN) 60°/sec	Average gain	49	0.39	0.18	300	0.61	0.15	0.000	0.4	0.9	25	51.0%	0.000	14	43.8%	0.000
	Area under fit (AUF) mean	49	7,667	1866	300	8,141	1,404	0.094	6,262	n/a	12	24.5%	0.000	6	18.8%	0.002
Gaze Horizontal (GH), in dark	PSPV	52	−0.43	1.18	—	—	—	—	−1.0	1.0	22	42.3%	—	16	45.7%	—
	Number of beats	52	7.52	5.96	—	—	—	—	n/a	4	35	67.3%	—	23	65.7%	—
	Number of SWJ	52	6.79	6.08	—	—	—	—	n/a	5	26	50.0%	—	14	40.0%	—
Subjective Visual Vertical (SVV)	Overall error mean	50	−0.64	2.61	287	0.02	1.51	0.091	−2.96	2.96	10	20.0%	0.000	9	26.5%	0.000
Auditory Reaction Time (ART)	Mean latency	51	278	81	300	234	61	0.000	n/a	316	14	27.5%	0.000	6	18.2%	0.002
Visual Reaction Time (VRT)	Mean latency	50	295	74	300	274	37	0.053	n/a	343	10	20.0%	0.000	6	18.2%	0.002
Saccade and Reaction Time (SRT)	Saccadic metrics															
	Latency mean, rightward	52	0.25	0.07	300	0.20	0.04	0.000	n/a	0.29	4	7.7%	0.567	3	8.6%	0.561
	Final acc. Grand mean	52	95.0	13.5	—	—	—	—	79	106	12	23.1%	0.000	9	25.7%	0.000
	Motor metrics															
	Latency means, right button	50	0.60	0.21	300	0.50	0.12	0.002	n/a	0.65	16	32.0%	0.000	11	33.3%	0.000
Predictive Saccades (PS)	(R) % of predicted	52	26.2	25.9	300	65.4	22.5	0.000	17	n/a	24	46.2%	0.000	14	40.0%	0.000

Except for the Gaze test metrics and for the Initiation latency (SPH and SPV), the 95% RI limits are computed (with FDA approval) using a normative database that included both male and female volunteers, aged 18–45, who were free of any neurological, vestibular disorders, or other head injuries. The normative limits for Gaze are based on the researchers’ previous experience. n/a refers to a situation with no upper or lower RI limit.

**Table 4:** Logistic regression models: VM vs. healthy participants.

Tests	Metrics	N	Estimate	*p*	AUC	Somers’ D
Saccade— Random, Horizontal (SH)	Latency grand mean	352	67.0	*0.000*	0.96	0.92
	Accuracy grand mean	352	−0.058	*0.034*	0.88	0.75
	(RR) Accuracy % of undershoot	352	0.073	*0.000*	0.92	0.83
	Final accuracy grand mean	352	0.034	*0.438*	0.88	0.76
	(RR) Final acc. % of undershoot	352	0.190	*0.000*	0.95	0.90
	Area under fit (AUF) mean	352	−0.00003	*0.856*	0.88	0.75
Saccade— Random, Vertical (SV)	Latency grand mean	352	58.0	*0.000*	0.95	0.91
	Accuracy grand mean	352	−0.010	*0.600*	0.88	0.75
	(RU) Accuracy % of undershoot	352	0.013	*0.086*	0.88	0.76
	Final accuracy grand mean	352	0.045	*0.070*	0.89	0.77
	(RU) Final acc. % of undershoot	352	0.020	*0.069*	0.88	0.76
	Area under fit (AUF) mean	352	−0.00028	*0.053*	0.88	0.76
Smooth Pursuit— Horizontal (SPH) 0.1 Hz	Velocity gain rightward	352	−2.85	*0.119*	0.88	0.75
	Velocity saccade, %	352	0.094	*0.000*	0.91	0.83
	Position gain	352	−4.78	*0.343*	0.88	0.75
Smooth Pursuit— Horizontal (SPH) 0.75 Hz	Velocity gain rightward	352	−3.94	*0.003*	0.88	0.77
	Velocity saccade, %	352	0.056	*0.000*	0.91	0.81
	Position gain	352	−1.55	*0.320*	0.87	0.75
Smooth Pursuit—Vertical (SPV) 0.1 Hz	Velocity gain up	352	−1.63	*0.246*	0.88	0.76
	Velocity saccade, %	352	0.140	*0.000*	0.95	0.89
	Position gain	352	4.87	*0.100*	0.88	0.76
Smooth Pursuit—Vertical (SPV) 0.75 Hz	Velocity gain up	352	−2.20	*0.019*	0.88	0.77
	Velocity saccade, %	352	0.050	*0.000*	0.90	0.80
	Position gain	352	2.60	*0.071*	0.88	0.76
Optokinetic (OKN) 20°/sec	Average gain	350	−5.66	*0.001*	0.89	0.77
	Area under fit (AUF) mean	350	−0.00031	*0.018*	0.89	0.78
Optokinetic (OKN) 60°/sec	Average gain	349	−6.33	*0.000*	0.92	0.83
	Area under fit (AUF) mean	349	−0.00008	*0.563*	0.88	0.77
Subjective Visual Vertical (SVV)	Overall error mean	337	−0.154	*0.145*	0.88	0.76
Auditory Reaction Time (ART)	Mean latency	351	0.004	*0.129*	0.88	0.76
Visual Reaction Time (VRT)	Mean latency	350	0.000	*0.914*	0.88	0.76
Saccade and Reaction Time (SRT)	Saccadic metrics					
	Latency mean, rightward	352	26.7	*0.000*	0.92	0.84
	(LR) Acc. % of undershoot	332	0.033	*0.025*	0.87	0.74
	(RR) Acc. % of undershoot	332	0.024	*0.020*	0.87	0.74
	Motor metrics					
	Latency mean, right button	350	2.25	*0.087*	0.88	0.76
Predictive Saccades (PS)	(L) % of predicted	352	−0.056	*0.000*	0.93	0.87
	(R) % of predicted	352	−0.057	*0.000*	0.94	0.87

*N* represents the total number of subjects up to a maximum of 52 VM patients and 300 health subjects. Table entries are estimated values of the regression coefficients with corresponding *p*-values, the estimated value of the area under the receiver operating characteristic curve (AUC) statistics and the related Somers’ D statistics computed as 2·AUC – 1.

### 3.1 Oculomotor domain

Horizontal and vertical random saccade tests (SH and SV) demonstrated strong discrimination between VM patients and controls. Saccade latency (time from stimulus presentation until a saccade is initiated) was significantly prolonged in VM patients. The mean SH latency was 0.24 ± 0.04 s in VM patients compared with 0.18 ± 0.02 s in controls (*p* < 0.0001; Table 3). Relative to normative reference intervals, 54.3 and 51.4% of VM participants aged 18 to 45 (59.6% when all 52 patients aged 18 to 70 were considered) demonstrated abnormal latencies for SH and SV (*p* < 0.0001), respectively.

The logistic regression analyses demonstrated excellent discriminative ability for saccade latency metrics, with AUC values ranging from 0.95 to 0.96 and corresponding Somers’ D values from 0.91 to 0.92 (Table 4). These results indicate strong single-metric discrimination. It should be noted that, when age and gender are not considered, the AUC and Somers’ D values are ranging from 0.94 to 0.95 and 0.89 to 0.90, respectively. Additional saccade metrics, including main and final saccade accuracy, further improved discrimination. Horizontal saccade (SH) metrics were generally more informative than vertical metrics. Abnormal grand mean accuracy was observed in 31.4% (*p* < 0.0001) of SH responses (aged 18–70: 28.8%, *p* < 0.0001) compared with 11.4% (*p* = 0.175) for SV responses (aged 18–70: 15.4%, *p* = 0.002).

Accuracy expressed as the percentage of hypometric (undershoot) saccades demonstrated improved discriminative ability, particularly for final accuracy (AUC = 0.95 with Somers’ D = 0.90; when age and gender are not considered in the model, AUC = 0.89 with Somers’ D = 0.78). The area under the main sequence fit (AUF) metric was not informative for SH but showed discriminative value for SV, in particular, with AUC and Somers’ D estimated at 0.88 and 0.76, respectively (when age and gender are not considered, it shows a modest discriminatory ability: AUC = 0.66; Somers’ D = 0.31). Smooth pursuit testing revealed abnormalities in both horizontal (SPH) and vertical (SPV) tracking. At slow eye movement (stimulus frequencies 0.1 Hz, 10° amplitude), abnormal saccadic intrusion rates were observed in 42.9% of SPH and 62.9% of SPV responses of 18–45-year-olds. The SPV metric at 0.1 Hz demonstrated high discriminative ability (AUC = 0.95; Somers’ D = 0.89). Initiation latency was also prolonged in VM patients, particularly at higher stimulus frequencies (0.75 Hz).

Optokinetic nystagmus (OKN) testing revealed reduced gain and increased asymmetry in VM patients. Abnormally low gain was observed in 36.4% of participants aged 18–45 at 20°/s and 43.8% at 60°/s (*p* < 0.0001 for both). Discrimination was greater at 60°/s (AUC = 0.92 with Somers’ D = 0.83, and AUC = 0.84 with Somers’ D = 0.67 when age and gender are not considered) than at 20°/s.

### 3.2 Vestibular domain

Subjective visual vertical (SVV) testing revealed abnormal positional error (≥3°) in 18.2% (*p* = 0.002) of VM patients aged 18–45. Horizontal and vertical gaze tests (GH and GV) demonstrated increased nystagmus activity in the dark. Peak slow phase velocity exceeded ±1°/s in 45.7% (*p* < 0.0001) of participants aged 18–45. Elevated numbers of nystagmus beats and square-wave jerks were observed in 65.7 and 40% of participants, respectively.

### 3.3 Reaction time domain

Auditory and visual reaction time (ART and VRT) latencies were abnormal in 26.5 and 18.2% (*p* < 0.002) of VM patients aged 18–45, respectively. However, the findings in logistic regression indicate that when age and gender are considered, the effect of the metrics does not explain the difference between the two cohorts. Combined saccade and motor reaction time (SRT) testing revealed significantly prolonged motor response latencies in VM patients (*p* = 0.002), with abnormal rates of 33.3% (right) and 39.4% (left). Motoric latency (right) and saccadic accuracy undershoot in the SRT test demonstrated high discriminative ability, AUC = 0.92 and 0.87 (AUC = 0.92 and 0.76 without controlling for age and gender), respectively.

### 3.4 Cognitive domain

**Figure 3:**
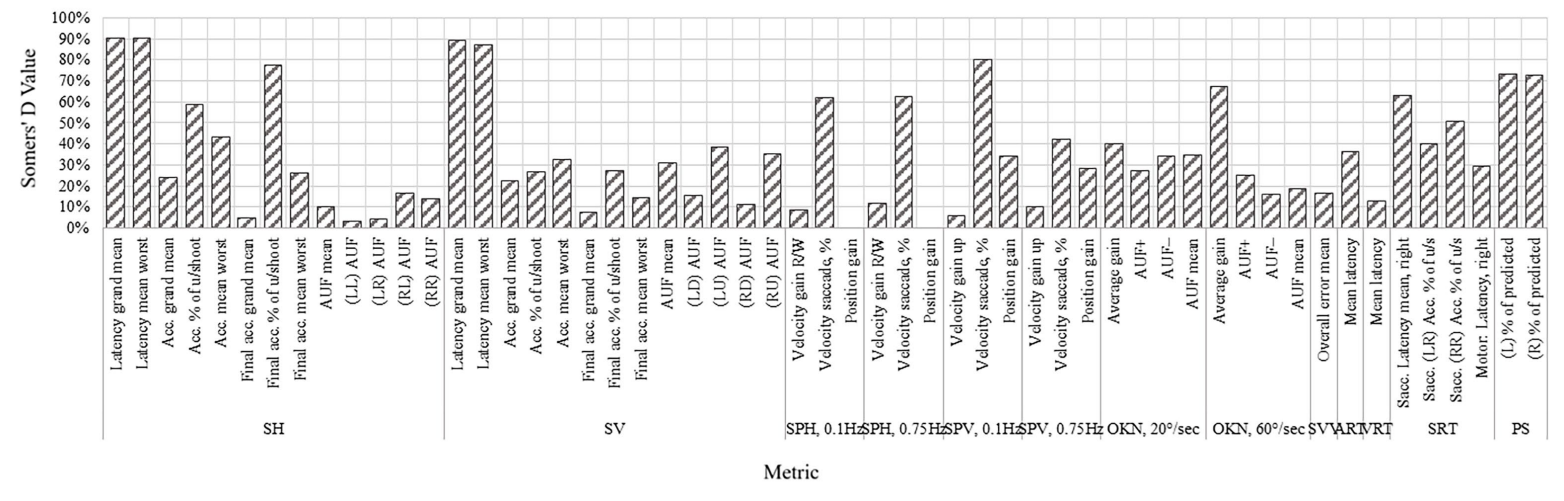
Percentage of subjects outside normative range for each of the metrics (for all age groups).

**Figure 4:**
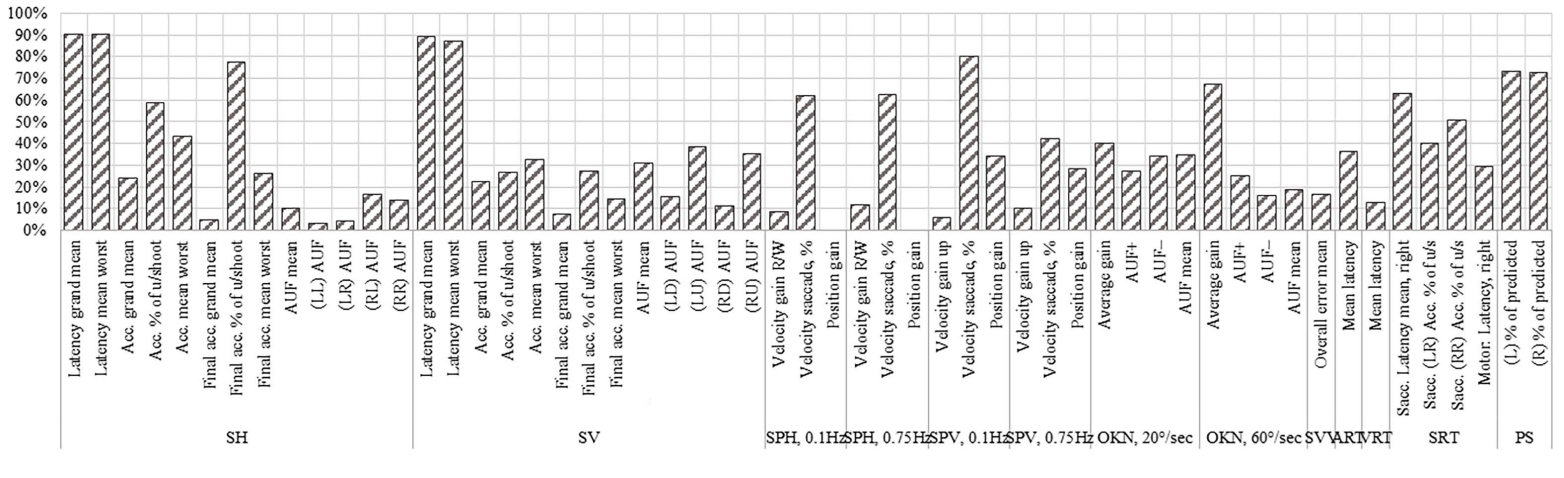
Somers’ D values for each of the metrics without age and gender adjustment.

The proportion of correctly predicted saccades was significantly reduced in VM patients compared with controls (*p* < 0.0001), with abnormal performance observed in 31.4–40.0% (*p* < 0.0001) of participants aged 18–45. This metric demonstrated strong discriminative performance with AUC of 0.93–0.94 (AUC of 0.86–0.87 without controlling for age and gender). Figures 3, 4 summarize abnormal rates and Somers’ D values for key OVRT-C metrics.

### 3.5 Multiple model

Stepwise multiple logistic regression identified six OVRT-C metrics that best differentiated VM patients from controls (Table 5). The model demonstrated excellent performance (AUC = 0.996), with specificity of 0.959 and sensitivity of 0.997 (at a cutoff value of 0.5), with relatively narrow corresponding 95% confidence intervals (see Supplementary Table S3). When comparing the original results (for all observations) with the results obtained using leave-one-out cross-validation, it can be observed that the statistics and corresponding confidence intervals are relatively stable (given that the sample is relatively small, especially the VM participants compared to the healthy controls), indicating of well-specified model without appreciable overfitting problem.

**Table 5:** Multiple logistic regression model: VM vs. healthy participants.

Test	Metrics	Estimate	*p*
Intercept		−21.61	0.004
Saccade – Random, Horizontal (SH)	Final accuracy, percentage of under shoot	0.24	0.004
Saccade – Random, Vertical (SV)	Latency grand mean (sec)	53.87	0.012
Smooth Pursuit – Vertical (SPV), 0.1 Hz	Velocity saccade (%)	0.20	0.001
Optokinetic (OKN), 20°/sec	Average gain	15.87	0.038
Optokinetic (OKN), 60°/sec	Average gain	−13.28	0.006
Predictive Saccades (PS)	Percentage of predicted saccades	−0.11	0.001
Number of observations	(VM/Healthy)	349	(49/300)

### 3.6 Symptom and DHI correlations

We examined the distribution of vestibular migraine (VM) symptoms and common comorbidities and assessed whether their presence or absence was associated with OVRT-C test performance using Spearman’s rank correlation coefficient. Symptom frequencies and percentages are summarized in Table 2 and comorbidities in Table 6. The most frequently reported symptoms were spontaneous internal vertigo (78.8%), visually induced vertigo (75.0%), and head motion–induced vertigo (63.5%). Correlations between symptom profiles and OVRT-C test metrics are presented in Supplementary Table S4.

**Table 6:** Number of patients who reported the disorders (comorbidities) prior to onset of vestibular symptoms and as current problems at time of testing.

	Prior		At time	
Disorders	*N*	%	*N*	%
Anxiety	25	48.1	33	63.5
Depression	18	34.6	21	40.4
Insomnia	20	38.5	29	44.8
Motion sickness	24	46.2	13	25.0

Reaction time domain measures, including auditory reaction time (ART), visual reaction time (VRT), and saccade–reaction time (SRT), demonstrated associations with visually induced vertigo, head motion–induced vertigo, and dizziness accompanied by nausea. Specifically, ART and VRT mean latency were positively correlated with head motion–induced vertigo, with Spearman’s rho values of 0.33 (*p* < 0.01) and 0.40 (*p* = 0.02), respectively. SRT metrics, particularly motor response latency, were associated with both visually induced vertigo and head motion–induced vertigo.

As shown in Supplementary Table S5, patients reporting vomiting exhibited higher vertical smooth pursuit (SPV) velocity gain at 0.1 Hz (rho = 0.36; *p* = 0.01) and greater horizontal saccade (SH) accuracy (e.g., accuracy grand mean: rho = 0.30; *p* = 0.03). An unexpected finding was that VM patients reporting visual snow demonstrated a higher percentage of correctly predicted saccades (rho = 0.27; *p* = 0.04). In addition, increased optokinetic nystagmus (OKN) average gain was associated with tinnitus, particularly during the 60°/s test (rho = 0.36; *p* = 0.01).

Participants were also evaluated for a history of anxiety, depression, insomnia, and motion sickness, which are common comorbidities in VM. Frequencies of these conditions prior to VM onset and at the time of testing are reported in Table 6. As shown in Supplementary Table S6, VM patients with anxiety or motion sickness tended to demonstrate reduced SH and vertical saccade (SV) accuracy. Increased SPV velocity gain at both 0.1 Hz and 0.75 Hz was associated with motion sickness (*p* = 0.01) and showed a trend toward association with insomnia (0.05 < *p* ≤ 0.10).

All participants except one completed the Dizziness Handicap Inventory (DHI) (Table 7). Based on total DHI scores, participants were categorized as having no, mild, moderate, or severe handicap, with the largest proportion (33.3%) classified as having severe handicap. Correlations between DHI scores and OVRT-C metrics are summarized in Supplementary Table S7. Higher total DHI scores were associated with increased saccadic intrusions during slow-speed horizontal smooth pursuit (rho = 0.37–0.38; *p* = 0.01) and with reduced SH accuracy. The strongest relationships were observed within the functional DHI domain, particularly with SH accuracy metrics, SV latency measures, and slow-speed horizontal smooth pursuit parameters.

**Table 7:** Classification of patients into four severity groups according to DHI score (left); mean and standard deviation (SD) of total DHI scores and their domain (right).

Severity classification	Score	*N*	%	DHI score	Mean	SD
No handicap	0–15	3	5.9	Total	43.5	19.5
Mild handicap	16–34	16	31.4	Domain		
Moderate handicap	35–52	15	29.4	Physical	12.7	6.3
Severe handicap	54–100	17	33.3	Emotional	14.7	7.1
Total		51	100.0	Functional	16.1	8.7

## 4 Discussion

Vestibular migraine (VM) is a disabling and frequently underdiagnosed migraine subtype for which no objective diagnostic test currently exists. The primary objective of this study was to identify a constellation of abnormalities on oculomotor, vestibular, reaction time, and cognitive (OVRT-C) testing that could aid in the diagnosis of VM. Prior studies using vestibular and oculomotor testing have demonstrated abnormalities in subsets of VM patients. For example, retrospective analysis of Videonystagmography (VNG) data identified vertical saccadic pursuit in approximately 40% of VM patients (11) while other studies have reported abnormalities such as nystagmus, impaired smooth pursuit, saccadic dysmetria, and asymmetric optokinetic nystagmus (OKN) in 42–92% of cases (12). Collectively, these findings support the presence of oculomotor and vestibular dysfunction in VM but highlight variability in test sensitivity across methodologies (13).

Using OVRT-C testing, we developed a multiple logistic regression model incorporating six metrics—horizontal saccade (SH) accuracy (percentage undershoot), vertical saccade (SV) latency, vertical smooth pursuit (SPV) saccadic component, OKN average gain at 20°/s and 60°/s, and percentage of correctly predicted saccades—that demonstrated excellent discrimination between VM patients and healthy controls. The “typical” VM patient in this cohort exhibited hypometric horizontal saccades, prolonged vertical saccade latency, increased saccadic intrusions during smooth pursuit (particularly vertical), reduced OKN gain at higher stimulus velocities, and impaired anticipatory saccade generation. Together, these findings suggest deficits across multiple eye movement subsystems rather than impairment localized to a single pathway. The resulting multiple model demonstrated very high diagnostic performance (AUC = 0.996), with high sensitivity (99.7%) and specificity (95.9%).

The neural circuitry underlying saccades, smooth pursuit, and OKN is distributed and highly interconnected, involving cortical regions (frontal, parietal, temporal, and occipital lobes), the superior colliculus, pontine nuclei, periaqueductal gray matter, vestibulocerebellum, and optic motoneurons (21). Each eye movement modality relies on distinct yet overlapping networks. The presence of abnormalities across saccadic, pursuit, and optokinetic responses in VM supports the concept of widespread network dysfunction, consistent with current models of migraine as a disorder of altered sensory processing and impaired central integration rather than a focal deficit.

Abnormalities in subjective visual vertical (SVV) were observed in a subset of VM patients. Prior literature on SVV in VM is mixed, with some studies demonstrating abnormalities (14) and others reporting limited diagnostic utility (11, 22). A Meta-analysis of 7 studies with 816 subjects with either migraine or tension-type headache found that both groups demonstrated moderately abnormal SVV results (23). A recent study found that SVV was of low diagnostic value in VM overall, but also noted substantial individual variation in SVV results (24). Our findings are consistent with the notion that SVV abnormalities may characterize a specific VM phenotype, potentially related to altered graviceptive processing or tilt perception, rather than representing a universal feature of the disorder.

Reaction time abnormalities were present in a smaller proportion of patients when assessed in isolation; however, when reaction time tasks were combined with saccadic demands, deficits became more prominent. VM patients demonstrated increased saccade latency and reduced accuracy, particularly during dual-task paradigms. These findings may reflect the cognitive slowing or “brain fog” frequently reported by VM patients. Importantly, abnormalities emerged under increased task complexity, suggesting reduced cognitive reserve or impaired integration of motor and sensory processes. Consistent with this interpretation, several OVRT-C metrics—particularly those combining saccadic and reaction time components—were correlated with the functional domain of the Dizziness Handicap Inventory (DHI), which captures difficulty performing complex occupational, household, and social activities. OVRT-C testing may therefore serve as an objective biomarker of functional impairment that is often difficult for patients to articulate.

This study demonstrates that OVRT-C testing can be performed in approximately 15 min using a single testing platform, without head movement or positional maneuvers that are often poorly tolerated by VM patients. The metrics generated are objective, quantifiable, and readily interpretable, offering clinically useful data to support diagnostic decision-making.

Several limitations should be acknowledged. First, only VM patients were studied; thus, we cannot determine whether this constellation of OVRT-C abnormalities is specific to VM or shared with other migraine phenotypes. Previous studies have shown vestibular and oculomotor abnormalities in migraine patients without vertigo, suggesting partial overlap (10). Second, we cannot assess the specificity of this battery for VM relative to other vestibular or neurologic disorders. Third, patients were tested irrespective of attack status, and abnormalities specific to the actual state may not have been captured. Most participants were receiving migraine preventive therapy, which may have influenced test performance; however, this reflects real-world clinical practice. The cohort was predominantly white and female and recruited from a tertiary headache center, potentially limiting generalizability. Finally, although nonresponse bias is possible, only two patients declined participation due to symptom concerns, making significant impact unlikely.

Future studies should evaluate OVRT-C testing in other vestibular and neurologic populations to determine diagnostic specificity, and in migraine patients without vestibular symptoms to better delineate shared versus distinct pathophysiologic mechanisms. Larger cohorts may also clarify whether distinct VM phenotypes exhibit unique OVRT-C profiles and whether such profiling could guide individualized treatment strategies.

## 5 Conclusion

This study demonstrates that multiple OVRT-C metrics are strongly associated with vestibular migraine and that a concise battery of eye movement, vestibular, reaction time, and cognitive measures can reliably distinguish VM patients from healthy controls.

Key findings include:

- Prolonged horizontal and vertical saccade latency.
- Hypometric horizontal saccades and reduced accuracy.
- Increased saccadic intrusions during vertical smooth pursuit.
- Reduced optokinetic nystagmus gain, particularly at 60°/s.
- Abnormal spontaneous nystagmus and square-wave jerks during dark gaze testing.
- Abnormal reaction times, particularly in combined saccade–reaction time tasks.
- OVRT-C metrics correlated with functional impairment on the Dizziness Handicap Inventory.

Collectively, these findings support the presence of widespread oculomotor, vestibular, and cognitive network dysfunction in VM and highlight the potential of OVRT-C testing as an objective diagnostic adjunct.

## Data Availability

The raw data supporting the conclusions of this article will be made available by the authors, without undue reservation.

## Glossary

OVRT-C: Oculomotor, Vestibular, Reaction Time, and Cognitive
VM: Vestibular Migraine
ICHD-3: International Classification of Headache Disorders, 3rd edition
VNG: Videonystagmography
SVV: Subjective Visual Vertical
VEMP: Vestibular-Evoked Myogenic Potential
DHI: Dizziness Handicap Inventory
AUC: Area Under the Receiver Operating Characteristic Curve
LOOCV: Leave-One-Out Cross-Validation
SH: Horizontal Saccade
SV: Vertical Saccade
AUF: Area Under Main Sequence Fit
SPH: Horizontal Smooth Pursuit
SPV: Vertical Smooth Pursuit
OKN: Optokinetic Nystagmus
GH: Horizontal Gaze
GV: Vertical Gaze
PSPV: Peak Slow Phase Velocity
SWJ: Square-Wave Jerk
ART: Auditory Reaction Time
VRT: Visual Reaction Time
SRT: Saccade Reaction Time
PS: Predicted Saccades
