# Supplementary material for: Objective oculomotor, vestibular, reaction time, and cognitive signatures of vestibular migraine
Source: Front Neurol. 2026 May 20;17:1789811. doi: 10.3389/fneur.2026.1789811 (PMC13229772; doi:10.3389/fneur.2026.1789811)
Supplement: Supplementary file 13 [file Supplementary_file_1.DOCX]

**Supplementary Materials to**

**“Objective Oculomotor, Vestibular, Reaction Time, and Cognitive Signatures of Vestibular Migraine”**

Claire E. J. Ceriani, MD^1^, Alexandr Braverman, PhD^2^, Alexander Kiderman, PhD^2^

^1^ Thomas Jefferson University, Philadelphia, PA, United States

^2^ Spryson America, Inc., Pittsburgh, PA, United States

**Table S.1. Battery of OVRT-C Tests and Metrics Measured for Each Test**

| **Test** | **Metrics** |
| --- | --- |
| Saccade – Random, Horizontal (SH): subject follows a dot displayed 30 times at pseudo-randomly distributed times (between 1 to 2 seconds) and pseudo-random displacements on either a horizontal plane (-30 to +30 degrees). | - - - 1. Latency (sec) = time from stimulus presentation until a saccade is initiated. Data are presented as an average of all saccade onset latencies.       2. Accuracy (%) = difference between eye position and stimulus position for the main saccade, expressed in percentage relative to stimulus position. Data are presented as an average of all main saccade accuracies.       3. Final accuracy (%) = difference between eye position and stimulus position for the final position, including corrective saccades, expressed in percentage relative to stimulus position. Data are presented as an average of all saccade accuracies.       4. Area under main sequence fit (AUF) (deg^2^/sec). Eye velocity is plotted as a function of saccade displacement and fitted with an exponential function. To evaluate the overall velocity and amplitude relationship, the software computes the area under the curve, out to 30 degrees of eye displacement = AUF. |
| Saccade – Random, Vertical (SV): subject follows a dot displayed 30 times at pseudo-randomly distributed times (between 1 to 2 seconds) and pseudo-random displacements on either a vertical plane (-20 to +20 degrees). | See previous. |
| Smooth Pursuit: subject follows a dot as it displaced (moves) sinusoidally horizontally then vertically at different speeds: Smooth Pursuit – Horizontal (SPH) 0.1 Hz, 3 cycles; 0.75 Hz, 6 cycles. | 1. Velocity gain = ratio between the slow phase component of eye velocity and pursuit tracker stimuli. Data are presented separately for the leftward and rightward moving stimuli. 2. Asymmetry = represents the difference between gain calculated for leftward and rightward moving stimuli. 3. Position gain = ratio between eye and stimulus position. 4. Velocity saccade (saccadic component) (%) = percentage of eye movement spent on a saccadic movement versus pursuit movement. 5. Initiation latency (msec) = time from stimulus presentation until a smooth pursuit movement is initiated. |
| Smooth Pursuit – Vertical (SPV) 0.1 Hz, 3 cycles; 0.75 Hz, 6 cycles. | See previous. |
| Optokinetic (OKN) 20 deg/sec: subjects see a field of dots moving on the display first to the right, then to the left, with eye tracking throughout the test with a velocity of 20 deg/sec. Each test consists of a stimulus rotating for 10 seconds clockwise (CW) and then 10 seconds counterclockwise (CCW), with 3 seconds of rest between CW and CCW rotation. | 1. Average eye velocity CW and CCW (deg/sec) = eye velocity during the slow phase of nystagmus for stimuli moving in clockwise (CW) and counterclockwise (CCW) direction. 2. Gain = ratio between average eye slow phase and stimulus velocity for CW and CCW segments. 3. Gain asymmetry (%) = represents the difference between gain calculated for CW and CCW segments. 4. Area under main sequence fit (AUF) (deg^2^/sec). Fast phase of OKN nystagmus beats is plotted as a function of the beats length and fitted with an exponential function. To evaluate the overall velocity and amplitude relationship, the software computes the area under the curve = AUF for CW and CCW stimulus movement. |
| Optokinetic (OKN) 60 deg/sec | See previous. |
| Gaze Horizontal (GH): subject fixates a light stimulus placed on 15 deg from the center to the left and then to the right of the screen for 10 seconds. The light is turned off and the subject is required to continue to fixate at the spot where the light was for 15 seconds. | Direction and velocity of nystagmus beats and number of square wave jerks (SWJ) for horizontal and vertical nystagmus during fixation and in the dark with gaze to the left and right. Average and peak slow phase velocity (ASPV and PSPV) computed for horizontal and vertical eye movement components with and without fixation. |
| Gaze Vertical (GV) | See previous. |
| Subjective Visual – Vertical (SVV): subject is presented with a non-vertical line and by using the left and right buttons on the handheld control box, orient the line to the vertical (upright) position, and then press the accept button on the control box. | Mean error (deg) = difference between subject’s orientation angle and true vertical. Data are presented as a mean of errors of all measurements. |
| Auditory Reaction Time (ART): 20-25 sound stimuli are presented with a random timing. The subject is directed to signal their recognition by pressing a button. | Latency (msec) = time difference from stimulus presentation until button is pressed. |
| Visual Reaction Time (VRT): 20-25 light stimuli are presented in the center of the screen, with a random timing. The subject is directed to signal their recognition by pressing a button. | Latency (msec) = time difference from stimulus presentation until button is pressed. |
| Saccade and Reaction Time (SRT): 30 visual saccadic stimuli are randomly projected every 1 to 2 seconds with a displacement of -30 to +30 degrees. The subjects are directed to gaze at the dot (saccadic stimulus) and then press either the left or right button to record whether the stimulus was projected to the right or to the left. | *Saccadic stimulus:*   1. Latency (sec) 2. Accuracy (%) 3. Final Accuracy (%)   (The metrics are defined the same as for SH.)  *Motor reaction time variables:*   1. Latency means (sec) – for left button = time difference from stimulus presentation until the left button is pressed. The same for ‘right button’. |
| Predictive Saccades (PS): subject is directed to follow a dot as it is displayed. Subject is presented with 6 pseudo-random saccade stimuli followed by 21 mirrored saccade stimuli with repeated displacement ±10 degrees, horizontal, at a constant time interval of 0.65 seconds. | Percentage of correctly predicted saccades. |
|  |  |

**Table S.2. Abnormal Rates Among VM Participants and Student’s t-Test to Evaluate the Mean Difference Between VM and Healthy** **Participants**

| **Tests** | **Metrics** |  | **t-Test** (Mean Difference) | | | | | | | | |  | **Abnormal Rate** | | | | | | | | | |
| --- | --- | --- | --- | --- | --- | --- | --- | --- | --- | --- | --- | --- | --- | --- | --- | --- | --- | --- | --- | --- | --- | --- |
|  |  |  | **VM** | | |  | **Healthy** | | |  | ***p*** |  | **95% RI limits** | |  | **VM (All)** | | |  | **VM (Age ≤ 45)** | | |
|  |  |  | **N** | **Mean** | **SD** |  | **N** | **Mean** | **SD** |  | (*Mean*  *Diff*) |  | **Lower** | **Upper** |  | **N**  **Abn** | **%**  **Abn** | ***p*** |  | **N**  **Abn** | **%**  **Abn** | ***p*** |
|  |  |  |  |  |  |  |  |  |  |  |  |  |  |  |  |  |  |  |  |  |  |  |
| **Saccade**— **Random, Horizontal (SH)** | Latency grand mean |  | 52 | 0.24 | 0.04 |  | 300 | 0.18 | 0.02 |  | *0.000* |  | n/a | 0.22 |  | 31 | 59.6% | *0.000* |  | 19 | 54.3% | *0.000* |
|  | Accuracy grand mean |  | 52 | 89.0 | 10.4 |  | 300 | 92.5 | 5.6 |  | *0.020* |  | 81 | 103 |  | 15 | 28.8% | *0.000* |  | 11 | 31.4% | *0.000* |
|  | (RR) Acc. % of undershoot |  | 52 | 31.8 | 26.7 |  | 300 | 6.6 | 10.9 |  | *0.000* |  | — | — |  | — | — | — |  | — | — | — |
|  | Final accuracy grand mean |  | 52 | 96.0 | 6.1 |  | 300 | 96.3 | 3.6 |  | *0.711* |  | 89 | 104 |  | 10 | 19.2% | *0.000* |  | 9 | 25.7% | *0.000* |
|  | (RR) Final acc. % of u-shoot |  | 52 | 25.9 | 18.3 |  | 300 | 2.0 | 4.3 |  | *0.000* |  | — | — |  | — | — | — |  | — | — | — |
|  | Area under fit (AUF) mean |  | 52 | 10167 | 1312 |  | 300 | 10357 | 1026 |  | *0.325* |  | 8239 | n/a |  | 4 | 7.7% | *0.567* |  | 3 | 8.6% | *0.561* |
| **Saccade**— **Random, Vertical (SV)** | Latency grand mean |  | 52 | 0.25 | 0.03 |  | 300 | 0.19 | 0.02 |  | *0.000* |  | n/a | 0.23 |  | 31 | 59.6% | *0.000* |  | 18 | 51.4% | *0.000* |
|  | Accuracy grand mean |  | 52 | 88.9 | 12.6 |  | 300 | 92.7 | 9.5 |  | *0.042* |  | 75 | 109 |  | 8 | 15.4% | *0.002* |  | 4 | 11.4% | *0.175* |
|  | (RU) Acc. % of undershoot |  | 52 | 30.8 | 27.9 |  | 300 | 18.6 | 22.3 |  | *0.004* |  | — | — |  | — | — | — |  | — | — | — |
|  | Final accuracy grand mean |  | 52 | 95.2 | 9.0 |  | 300 | 94.2 | 7.4 |  | *0.461* |  | 79 | 107 |  | 6 | 11.5% | *0.065* |  | 5 | 14.3% | *0.033* |
|  | (RU) Final acc. % of u-shoot |  | 52 | 16.4 | 17.8 |  | 300 | 8.2 | 14.4 |  | *0.002* |  | — | — |  | — | — | — |  | — | — | — |
|  | Area under fit (AUF) mean |  | 52 | 8832 | 1472 |  | 300 | 9684 | 1339 |  | *0.000* |  | 7630 | n/a |  | 9 | 17.3% | *0.000* |  | 6 | 17.1% | *0.004* |
| **Smooth Pursuit**— **Horizontal (SPH) 0.1Hz** | Velocity gain rightward |  | 52 | 0.93 | 0.11 |  | 300 | 0.95 | 0.08 |  | *0.122* |  | 0.78 | 1.07 |  | 4 | 7.7% | *0.567* |  | 4 | 11.4% | *0.175* |
|  | Velocity gain leftward |  | 52 | 0.94 | 0.09 |  | — | — | — |  | — |  | 0.78 | 1.07 |  | 2 | 3.8% | *1.000* |  | 2 | 5.7% | *0.846* |
|  | Velocity gain asymmetry |  | 52 | -0.86 | 7.42 |  | — | — | — |  | — |  | -8.80 | 7.53 |  | 4 | 7.7% | *0.567* |  | 4 | 11.4% | *0.175* |
|  | Velocity saccade, % |  | 52 | 33.6 | 14.3 |  | 300 | 18.0 | 10.2 |  | *0.000* |  | n/a | 35 |  | 23 | 44.2% | *0.000* |  | 15 | 42.9% | *0.000* |
|  | Position gain |  | 52 | 1.00 | 0.06 |  | 300 | 1.00 | 0.02 |  | *0.567* |  | 0.96 | 1.04 |  | 11 | 21.2% | *0.000* |  | 10 | 28.6% | *0.000* |
|  | Initiation latency |  | 52 | 284 | 81 |  | — | — | — |  | — |  | n/a | 335 |  | 13 | 25.0% | *0.000* |  | 8 | 22.9% | *0.000* |
| **Smooth Pursuit**— **Horizontal (SPH) 0.75Hz** | Velocity gain rightward |  | 52 | 0.87 | 0.21 |  | 300 | 0.95 | 0.10 |  | *0.010* |  | 0.62 | 1.08 |  | 10 | 19.2% | *0.000* |  | 7 | 20.0% | *0.000* |
|  | Velocity gain leftward |  | 52 | 0.87 | 0.17 |  | — | — | — |  | — |  | 0.62 | 1.08 |  | 7 | 13.5% | *0.013* |  | 7 | 20.0% | *0.000* |
|  | Velocity gain asymmetry |  | 52 | -0.65 | 10.07 |  | — | — | — |  | — |  | -8.93 | 9.0 |  | 16 | 30.8% | *0.000* |  | 11 | 31.4% | *0.000* |
|  | Velocity saccade, % |  | 52 | 30.9 | 14.2 |  | 300 | 15.8 | 11.5 |  | *0.000* |  | n/a | 37 |  | 17 | 32.7% | *0.000* |  | 8 | 22.9% | *0.000* |
|  | Position gain |  | 52 | 0.93 | 0.17 |  | 300 | 0.96 | 0.07 |  | *0.280* |  | 0.79 | 1.10 |  | 13 | 25.0% | *0.000* |  | 10 | 28.6% | *0.000* |
|  | Initiation latency |  | 52 | 268 | 60 |  | — | — | — |  | — |  | n/a | 252 |  | 29 | 55.8% | *0.000* |  | 19 | 54.3% | *0.000* |
| **Smooth Pursuit**— **Vertical (SPV) 0.1Hz** | Velocity gain up |  | 52 | 0.88 | 0.17 |  | 300 | 0.90 | 0.11 |  | *0.515* |  | 0.69 | 1.07 |  | 11 | 21.2% | *0.000* |  | 6 | 17.1% | *0.004* |
|  | Velocity gain down |  | 52 | 0.91 | 0.19 |  | — | — | — |  | — |  | 0.69 | 1.07 |  | 6 | 11.5% | *0.065* |  | 5 | 14.3% | *0.033* |
|  | Velocity gain asymmetry |  | 52 | -1.16 | 11.98 |  | — | — | — |  | — |  | -12.36 | 11.46 |  | 15 | 28.8% | *0.000* |  | 11 | 31.4% | *0.000* |
|  | Velocity saccade, % |  | 52 | 39.40 | 17.76 |  | 300 | 14.1 | 8.5 |  | *0.000* |  | n/a | 32 |  | 32 | 61.5% | *0.000* |  | 22 | 62.9% | *0.000* |
|  | Position gain |  | 52 | 1.02 | 0.13 |  | 300 | 0.99 | 0.03 |  | *0.231* |  | 0.95 | 1.07 |  | 26 | 50.0% | *0.000* |  | 21 | 60.0% | *0.000* |
|  | Initiation latency |  | 52 | 282.3 | 90.3 |  | — | — | — |  | — |  | n/a | 311 |  | 16 | 30.8% | *0.000* |  | 14 | 40.0% | *0.000* |
| **Smooth Pursuit**— **Vertical (SPV) 0.75Hz** | Velocity gain up |  | 52 | 0.75 | 0.26 |  | 300 | 0.81 | 0.18 |  | *0.127* |  | 0.42 | 1.09 |  | 10 | 19.2% | *0.000* |  | 7 | 20.0% | *0.000* |
|  | Velocity gain down |  | 52 | 0.69 | 0.25 |  | — | — | — |  | — |  | 0.42 | 1.09 |  | 8 | 15.4% | *0.002* |  | 5 | 14.3% | *0.033* |
|  | Velocity gain asymmetry |  | 52 | 3.15 | 23.42 |  | — | — | — |  | — |  | -23.43 | 29.01 |  | 16 | 30.8% | *0.000* |  | 8 | 22.9% | *0.000* |
|  | Velocity saccade, % |  | 52 | 38.9 | 16.1 |  | 300 | 26.9 | 14.5 |  | *0.000* |  | n/a | 52 |  | 12 | 23.1% | *0.000* |  | 7 | 20.0% | *0.000* |
|  | Position gain |  | 52 | 0.98 | 0.20 |  | 300 | 0.91 | 0.11 |  | *0.016* |  | 0.73 | 1.11 |  | 15 | 28.8% | *0.000* |  | 8 | 22.9% | *0.000* |
|  | Initiation latency |  | 52 | 277 | 81 |  | — | — | — |  | — |  | n/a | 230 |  | 34 | 65.4% | *0.000* |  | 24 | 68.6% | *0.000* |
| **Optokinetic (OKN) 20°/sec** | Average gain |  | 50 | 0.77 | 0.14 |  | 300 | 0.86 | 0.08 |  | *0.000* |  | 0.66 | 0.97 |  | 15 | 30.0% | *0.000* |  | 12 | 36.4% | *0.000* |
|  | Asymmetry |  | 50 | 4.54 | 14.30 |  | — | — | — |  | — |  | -7.66 | 10.55 |  | 16 | 32.0% | *0.000* |  | 11 | 33.3% | *0.000* |
|  | Area under fit (AUF) mean |  | 50 | 7314 | 1636 |  | 300 | 8274 | 1447 |  | *0.000* |  | 5735 | n/a |  | 11 | 22.0% | *0.000* |  | 6 | 18.2% | *0.002* |
| **Optokinetic (OKN) 60°/sec** | Average gain |  | 49 | 0.39 | 0.18 |  | 300 | 0.61 | 0.15 |  | *0.000* |  | 0.4 | 0.9 |  | 25 | 51.0% | *0.000* |  | 14 | 43.8% | *0.000* |
|  | Asymmetry |  | 49 | 1.72 | 23.80 |  | — | — | — |  | — |  | -14.54 | 18.1 |  | 15 | 30.6% | *0.000* |  | 9 | 25.7% | *0.000* |
|  | Area under fit (AUF) mean |  | 49 | 7667 | 1866 |  | 300 | 8141 | 1404 |  | *0.094* |  | 6262 | n/a |  | 12 | 24.5% | *0.000* |  | 6 | 18.8% | *0.002* |
| **Gaze Horizontal (GH**),  in dark | PSPV |  | 52 | -0.43 | 1.18 |  | — | — | — |  | — |  | -1.0 | 1.0 |  | 22 | 42.3% | — |  | 16 | 45.7% | — |
|  | Number of beats |  | 52 | 7.52 | 5.96 |  | — | — | — |  | — |  | n/a | 4 |  | 35 | 67.3% | — |  | 23 | 65.7% | — |
|  | Number of SWJ |  | 52 | 6.79 | 6.08 |  | — | — | — |  | — |  | n/a | 5 |  | 26 | 50.0% | — |  | 14 | 40.0% | — |
| **Gaze Vertical (GV)**,  in dark | PSPV |  | 52 | -0.02 | 1.29 |  | — | — | — |  | — |  | -1.0 | 1.0 |  | 16 | 30.8% | — |  | 13 | 37.1% | — |
|  | Number of beats |  | 52 | 4.56 | 6.03 |  | — | — | — |  | — |  | n/a | 4 |  | 20 | 38.5% | — |  | 15 | 42.9% | — |
|  | Number of SWJ |  | 52 | 1.69 | 2.25 |  | — | — | — |  | — |  | n/a | 5 |  | 4 | 7.7% | — |  | 3 | 8.6% | — |
| **Subjective Visual Vertical (SVV)** | Overall error mean |  | 50 | -0.64 | 2.61 |  | 287 | 0.02 | 1.51 |  | *0.091* |  | -2.96 | 2.96 |  | 10 | 20.0% | *0.000* |  | 9 | 26.5% | *0.000* |
| **Auditory Reaction Time (ART)** | Mean latency |  | 51 | 278 | 81 |  | 300 | 234 | 61 |  | *0.000* |  | n/a | 316 |  | 14 | 27.5% | *0.000* |  | 6 | 18.2% | *0.002* |
| **Visual Reaction Time (VRT)** | Mean latency |  | 50 | 295 | 74 |  | 300 | 274 | 37 |  | *0.053* |  | n/a | 343 |  | 10 | 20.0% | *0.000* |  | 6 | 18.2% | *0.002* |
| **Saccade and Reaction Time (SRT)** | *Saccadic metrics:* |  |  |  |  |  |  |  |  |  |  |  |  |  |  |  |  |  |  |  |  |  |
|  | Latency grand mean |  | 52 | 0.24 | 0.06 |  | — | — | — |  | — |  | n/a | 0.29 |  | 4 | 7.7% | *0.567* |  | 2 | 5.7% | *0.846* |
|  | Latency mean, rightward |  | 52 | 0.25 | 0.07 |  | 300 | 0.20 | 0.04 |  | *0.000* |  | n/a | 0.29 |  | 4 | 7.7% | *0.567* |  | 3 | 8.6% | *0.561* |
|  | Latency means, leftward |  | 52 | 0.23 | 0.06 |  | — | — | — |  | — |  | n/a | 0.29 |  | 3 | 5.8% | *0.949* |  | 2 | 5.7% | *0.846* |
|  | (LR) Acc. % of undershoot |  | 32 | 19.1 | 18.3 |  | 300 | 7.1 | 9.8 |  | *0.001* |  | — | — |  | — | — | — |  | — | — | — |
|  | (RR) Acc. % of undershoot |  | 32 | 30.6 | 27.6 |  | 300 | 10.4 | 14.6 |  | *0.000* |  | — | — |  | — | — | — |  | — | — | — |
|  | Final acc. grand mean |  | 52 | 95.0 | 13.5 |  | — | — | — |  | — |  | 79 | 106 |  | 12 | 23.1% | *0.000* |  | 9 | 25.7% | *0.000* |
|  | *Motor metrics:* |  |  |  |  |  |  |  |  |  |  |  |  |  |  |  |  |  |  |  |  |  |
|  | Latency means, right button |  | 50 | 0.60 | 0.21 |  | 300 | 0.50 | 0.12 |  | *0.002* |  | n/a | 0.65 |  | 16 | 32.0% | *0.000* |  | 11 | 33.3% | *0.000* |
|  | Latency means, left button |  | 50 | 0.60 | 0.21 |  | — | — | — |  | — |  | n/a | 0.65 |  | 21 | 42.0% | *0.000* |  | 13 | 39.4% | *0.000* |
| **Predictive Saccades (PS)** | (L) % of predicted |  | 52 | 26.5 | 24.9 |  | 300 | 65.3 | 22.7 |  | *0.000* |  | 17 | n/a |  | 23 | 44.2% | *0.000* |  | 11 | 31.4% | *0.000* |
|  | (R) % of predicted |  | 52 | 26.2 | 25.9 |  | 300 | 65.4 | 22.5 |  | *0.000* |  | 17 | n/a |  | 24 | 46.2% | *0.000* |  | 14 | 40.0% | *0.000* |
|  |  |  |  |  |  |  |  |  |  |  |  |  |  |  |  |  |  |  |  |  |  |  |
| ▪ Except for the Gaze test metrics and for the Initiation latency (SPH and SPV), the 95% RI limits are computed (with FDA approval) using a normative database that included both male and female volunteers, aged 18-45, who were free of any neurological, vestibular disorders, or other head injuries. The normative limits for Gaze are based on the researchers’ previous experience. ▪ n/a refers to a situation with no upper or lower RI limit. | | | | | | | | | | | | | | | | | | | | | | |

**Table S.3. Diagnostic Statistics for Multiple Logistic Regression Model**

| **Statistics** |  | **All Observations** | | |  | **Leave-One-Out Cross-Validation** | | |
| --- | --- | --- | --- | --- | --- | --- | --- | --- |
|  |  | **Estimate** | **95% CI** | |  | **Estimate** | **95% CI** | |
|  |  |  | **Lower** | **Upper** |  |  | **Lower** | **Upper** |
|  |  |  |  |  |  |  |  |  |
| AUC |  | 0.996 | 0.978 | 1.000 |  | 0.977 | 0.887 | 0.999 |
| Somers’ D |  | 0.993 | 0.956 | 1.000 |  | 0.955 | 0.774 | 0.997 |
| Sensitivity |  | 0.959 | 0.868 | 1.000 |  | 0.918 | 0.800 | 0.976 |
| Specificity |  | 0.997 | 0.983 | 1.000 |  | 0.987 | 0.967 | 0.997 |
| 1-Specificity |  | 0.003 | 0.000 | 0.017 |  | 0.013 | 0.003 | 0.033 |
| 1-Sensitivity |  | 0.041 | 0.000 | 0.132 |  | 0.082 | 0.024 | 0.200 |
|  |  |  |  |  |  |  |  |  |
| Bootstrap method with 2000 replicates used to calculate the 95% confidence intervals (95% CI). | | | | | | | | |

**Table S.4. Correlation Between the Vestibular Symptoms and OVRT-C Tests**

| **Tests** | **Metrics** |  | **N** |  | **Spontaneous internal vertigo** | | **Spontaneous external vertigo** | | **Positional vertigo** | | **Visual induced vertigo** | | **Head motion induced vertigo** | | **Head motion induced dizz. nausea** | |  |
| --- | --- | --- | --- | --- | --- | --- | --- | --- | --- | --- | --- | --- | --- | --- | --- | --- | --- |
|  |  |  |  |  | **Rho** | ***p*** | **Rho** | ***p*** | **Rho** | ***p*** | **Rho** | ***p*** | **Rho** | ***p*** | **Rho** | ***p*** |  |
|  |  |  |  |  |  |  |  |  |  |  |  |  |  |  |  |  |  |
| **SH** | Latency grand mean |  | 52 |  | -0.14 | *0.32* | -0.05 | *0.73* | 0.02 | *0.89* | -0.07 | *0.62* | 0.22 | *0.11* | 0.17 | *0.22* |  |
|  | Accuracy grand mean |  | 52 |  | -0.37 | ***0.01*** | 0.05 | *0.71* | -0.06 | *0.67* | 0.03 | *0.84* | 0.03 | *0.86* | 0.04 | *0.76* |  |
|  | (RR) Accuracy % of undershoot |  | 52 |  | 0.11 | *0.45* | 0.06 | *0.66* | 0.08 | *0.56* | 0.01 | *0.92* | 0.00 | *0.98* | -0.05 | *0.71* |  |
|  | Final accuracy grand mean |  | 52 |  | -0.23 | *0.10* | 0.07 | *0.64* | -0.16 | *0.26* | -0.02 | *0.88* | -0.14 | *0.33* | -0.03 | *0.82* |  |
|  | (RR) Final acc. % of undershoot |  | 52 |  | -0.06 | *0.68* | 0.16 | *0.25* | 0.23 | *0.11* | 0.18 | *0.21* | 0.04 | *0.76* | -0.05 | *0.73* |  |
|  | Final accuracy mean worst |  | 52 |  | -0.05 | *0.75* | -0.02 | *0.88* | -0.10 | *0.47* | -0.04 | *0.79* | 0.00 | *0.98* | 0.10 | *0.50* |  |
|  | Area under fit (AUF) mean |  | 52 |  | -0.14 | *0.31* | -0.07 | *0.60* | 0.20 | *0.15* | 0.12 | *0.40* | 0.12 | *0.39* | -0.03 | *0.83* |  |
| **SV** | Latency grand mean |  | 52 |  | -0.08 | *0.55* | 0.00 | *0.99* | 0.18 | *0.19* | 0.03 | *0.83* | 0.15 | *0.30* | 0.09 | *0.53* |  |
|  | Accuracy grand mean |  | 52 |  | -0.04 | *0.78* | -0.08 | *0.56* | -0.08 | *0.58* | -0.14 | *0.32* | 0.00 | *0.99* | -0.06 | *0.68* |  |
|  | (RU) Accuracy % of undershoot |  | 52 |  | 0.17 | *0.23* | -0.06 | *0.68* | 0.10 | *0.47* | 0.03 | *0.82* | 0.00 | *0.98* | 0.07 | *0.64* |  |
|  | Final accuracy grand mean |  | 52 |  | 0.27 | ***0.05*** | 0.07 | *0.60* | -0.02 | *0.86* | -0.15 | *0.30* | -0.12 | *0.38* | -0.19 | *0.17* |  |
|  | (RU) Final acc. % of undershoot |  | 52 |  | -0.03 | *0.82* | 0.13 | *0.36* | 0.05 | *0.70* | 0.22 | *0.12* | 0.00 | *0.98* | 0.10 | *0.47* |  |
|  | Area under fit (AUF) mean |  | 52 |  | -0.16 | *0.25* | -0.08 | *0.59* | 0.08 | *0.58* | 0.01 | *0.96* | 0.09 | *0.54* | -0.03 | *0.83* |  |
| **SPH, 0.1Hz** | Velocity gain rightward |  | 52 |  | -0.03 | *0.85* | -0.17 | *0.24* | -0.08 | *0.56* | 0.12 | *0.42* | 0.15 | *0.28* | -0.02 | *0.89* |  |
|  | Velocity gain leftward |  | 52 |  | 0.09 | *0.51* | -0.02 | *0.87* | 0.00 | *0.99* | 0.16 | *0.25* | 0.19 | *0.17* | 0.09 | *0.54* |  |
|  | Velocity saccade, % |  | 52 |  | -0.17 | *0.23* | 0.25 | *0.08* | 0.14 | *0.33* | -0.09 | *0.52* | -0.14 | *0.33* | 0.04 | *0.77* |  |
|  | Position gain |  | 52 |  | 0.09 | *0.51* | -0.17 | *0.23* | -0.30 | ***0.03*** | 0.02 | *0.87* | 0.11 | *0.42* | 0.08 | *0.59* |  |
|  | Initiation latency |  | 52 |  | 0.05 | *0.72* | -0.13 | *0.35* | 0.27 | ***0.05*** | -0.15 | *0.29* | -0.09 | *0.52* | -0.08 | *0.55* |  |
| **SPH, 0.75Hz** | Velocity gain rightward |  | 52 |  | -0.09 | *0.54* | -0.01 | *0.93* | 0.06 | *0.67* | -0.08 | *0.55* | 0.04 | *0.79* | 0.13 | *0.38* |  |
|  | Velocity gain leftward |  | 52 |  | -0.06 | *0.67* | -0.04 | *0.75* | -0.07 | *0.61* | 0.14 | *0.33* | 0.04 | *0.78* | 0.04 | *0.80* |  |
|  | Velocity saccade, % |  | 52 |  | 0.10 | *0.50* | -0.32 | ***0.02*** | 0.12 | *0.40* | -0.16 | *0.24* | -0.02 | *0.89* | 0.11 | *0.44* |  |
|  | Position gain |  | 52 |  | 0.11 | *0.44* | -0.23 | *0.10* | -0.08 | *0.56* | -0.17 | *0.22* | 0.03 | *0.86* | -0.07 | *0.64* |  |
|  | Initiation latency |  | 52 |  | 0.01 | *0.95* | -0.05 | *0.75* | 0.21 | *0.14* | 0.19 | *0.18* | 0.26 | *0.06* | 0.17 | *0.24* |  |
| **SPV, 0.1Hz** | Velocity gain up |  | 52 |  | -0.09 | *0.51* | 0.07 | *0.64* | -0.01 | *0.95* | 0.21 | *0.14* | 0.08 | *0.59* | -0.05 | *0.71* |  |
|  | Velocity gain down |  | 52 |  | -0.02 | *0.91* | 0.09 | *0.53* | 0.16 | *0.26* | 0.20 | *0.15* | 0.27 | ***0.05*** | -0.05 | *0.72* |  |
|  | Velocity saccade, % |  | 52 |  | 0.11 | *0.43* | -0.06 | *0.69* | 0.11 | *0.43* | -0.08 | *0.59* | -0.05 | *0.71* | -0.03 | *0.82* |  |
|  | Position gain |  | 52 |  | -0.02 | *0.91* | 0.25 | *0.08* | 0.25 | *0.07* | 0.17 | *0.23* | 0.19 | *0.18* | -0.15 | *0.30* |  |
|  | Initiation latency |  | 52 |  | -0.15 | *0.30* | 0.02 | *0.86* | 0.12 | *0.40* | 0.06 | *0.68* | -0.03 | *0.81* | -0.17 | *0.22* |  |
| **SPV, 0.75Hz** | Velocity gain up |  | 52 |  | 0.04 | *0.78* | 0.13 | *0.37* | -0.12 | *0.40* | -0.07 | *0.62* | -0.04 | *0.79* | 0.01 | *0.94* |  |
|  | Velocity gain down |  | 52 |  | 0.08 | *0.57* | -0.08 | *0.57* | 0.05 | *0.73* | 0.14 | *0.34* | 0.10 | *0.46* | 0.05 | *0.72* |  |
|  | Velocity saccade, % |  | 52 |  | -0.19 | *0.17* | 0.05 | *0.75* | 0.03 | *0.81* | -0.23 | *0.10* | -0.07 | *0.62* | 0.13 | *0.36* |  |
|  | Position gain |  | 52 |  | 0.04 | *0.76* | 0.06 | *0.68* | 0.00 | *0.99* | -0.20 | *0.15* | -0.11 | *0.45* | 0.11 | *0.43* |  |
|  | Initiation latency |  | 52 |  | 0.06 | *0.66* | -0.08 | *0.57* | -0.18 | *0.21* | 0.04 | *0.78* | -0.11 | *0.46* | 0.08 | *0.56* |  |
| **OKN, 20°/sec** | Average gain |  | 50 |  | 0.04 | *0.79* | 0.16 | *0.27* | -0.04 | *0.77* | 0.12 | *0.39* | -0.06 | *0.68* | 0.20 | *0.16* |  |
|  | Area under fit (AUF) mean |  | 50 |  | -0.13 | *0.39* | -0.07 | *0.65* | 0.20 | *0.17* | -0.14 | *0.34* | -0.08 | *0.60* | 0.06 | *0.68* |  |
| **OKN, 60°/sec** | Average gain |  | 49 |  | 0.12 | *0.39* | 0.16 | *0.27* | -0.02 | *0.87* | 0.11 | *0.43* | -0.08 | *0.59* | 0.09 | *0.52* |  |
|  | Area under fit (AUF) mean |  | 49 |  | -0.15 | *0.31* | -0.06 | *0.69* | 0.35 | ***0.01*** | -0.03 | *0.86* | 0.11 | *0.44* | 0.14 | *0.33* |  |
| **SVV** | Overall error mean |  | 50 |  | -0.01 | *0.94* | 0.03 | *0.82* | 0.20 | *0.17* | 0.11 | *0.46* | 0.05 | *0.73* | 0.11 | *0.47* |  |
| **ART** | Mean latency |  | 51 |  | 0.01 | *0.94* | 0.25 | *0.07* | -0.13 | *0.35* | -0.02 | *0.88* | 0.33 | ***0.02*** | 0.11 | *0.43* |  |
| **VRT** | Mean latency |  | 50 |  | -0.06 | *0.69* | 0.03 | *0.84* | -0.17 | *0.24* | 0.02 | *0.90* | 0.40 | ***0.00*** | 0.12 | *0.40* |  |
| **SRT** | *Saccadic metrics:* |  |  |  |  |  |  |  |  |  |  |  |  |  |  |  |  |
|  | Latency grand mean |  | 52 |  | -0.01 | *0.94* | -0.03 | *0.83* | 0.03 | *0.84* | -0.01 | *0.97* | 0.26 | *0.06* | 0.21 | *0.14* |  |
|  | Latency mean, rightward |  | 52 |  | 0.03 | *0.81* | -0.06 | *0.65* | -0.03 | *0.84* | -0.01 | *0.93* | 0.21 | *0.14* | 0.27 | *0.06* |  |
|  | Latency mean, leftward |  | 52 |  | -0.06 | *0.67* | 0.08 | *0.59* | 0.11 | *0.42* | -0.01 | *0.97* | 0.23 | *0.10* | 0.10 | *0.47* |  |
|  | (LR) Acc. % of undershoot |  | 32 |  | 0.09 | *0.63* | -0.06 | *0.73* | -0.05 | *0.79* | -0.05 | *0.80* | -0.15 | *0.42* | -0.16 | *0.37* |  |
|  | (RR) Acc. % of undershoot |  | 32 |  | 0.04 | *0.83* | -0.01 | *0.97* | 0.24 | *0.19* | 0.16 | *0.39* | 0.13 | *0.47* | -0.20 | *0.28* |  |
|  | Final acc. grand mean |  | 52 |  | -0.15 | *0.30* | -0.10 | *0.48* | 0.04 | *0.78* | -0.04 | *0.76* | -0.01 | *0.93* | 0.25 | *0.07* |  |
|  | *Motor metrics:* |  |  |  |  |  |  |  |  |  |  |  |  |  |  |  |  |
|  | Latency mean, right button |  | 50 |  | 0.07 | *0.64* | 0.11 | *0.43* | 0.03 | *0.83* | 0.27 | ***0.05*** | 0.29 | ***0.04*** | 0.17 | *0.25* |  |
|  | Latency mean, left button |  | 50 |  | -0.03 | *0.86* | 0.18 | *0.20* | 0.05 | *0.76* | 0.28 | ***0.05*** | 0.36 | ***0.01*** | 0.13 | *0.35* |  |
| **PS** | (L) % of predicted |  | 52 |  | -0.07 | *0.61* | 0.21 | *0.13* | -0.03 | *0.84* | -0.12 | *0.41* | -0.13 | *0.35* | -0.12 | *0.38* |  |
|  | (R) % of predicted |  | 52 |  | -0.09 | *0.55* | 0.15 | *0.29* | 0.03 | *0.85* | -0.15 | *0.29* | 0.01 | *0.95* | -0.04 | *0.79* |  |
|  |  |  |  |  |  |  |  |  |  |  |  |  |  |  |  |  |  |
| Spearman’s rank correlation coefficient (rho) is used to measure the relationship between test metrics and symptoms using the CORR function in MATLAB software. p-value is calculated under two-tail hypothesis. | | | | | | | | | | | | | | | | | |

**Table S.5. Correlation Between OVRT-C Tests and Related Symptoms**

| **Tests** | **Metrics** |  | **N** |  | **Vomiting** | | **Visual**  **lag** | | **Oscil-**  **lopsia** | | **Visual**  **snow** | | **Tinnitus** | | **Ear**  **pain** | | **Transient decreased hearing** | |
| --- | --- | --- | --- | --- | --- | --- | --- | --- | --- | --- | --- | --- | --- | --- | --- | --- | --- | --- |
|  |  |  |  |  | **Rho** | ***p*** | **Rho** | ***p*** | **Rho** | ***p*** | **Rho** | ***p*** | **Rho** | ***p*** | **Rho** | ***p*** | **Rho** | ***p*** |
|  |  |  |  |  |  |  |  |  |  |  |  |  |  |  |  |  |  |  |
| **SH** | Latency grand mean |  | 52 |  | -0.12 | *0.38* | 0.08 | *0.56* | 0.04 | *0.76* | 0.09 | *0.53* | -0.03 | *0.81* | 0.08 | *0.58* | -0.14 | *0.34* |
|  | Accuracy grand mean |  | 52 |  | 0.30 | ***0.03*** | -0.10 | *0.49* | -0.15 | *0.29* | 0.05 | *0.71* | 0.05 | *0.75* | 0.18 | *0.21* | -0.08 | *0.59* |
|  | (RR) Accuracy % of undershoot |  | 52 |  | -0.18 | *0.21* | -0.04 | *0.76* | 0.14 | *0.31* | -0.13 | *0.35* | -0.15 | *0.29* | -0.08 | *0.55* | 0.12 | *0.38* |
|  | Final accuracy grand mean |  | 52 |  | 0.20 | *0.16* | -0.20 | *0.15* | -0.26 | *0.06* | -0.05 | *0.73* | 0.07 | *0.63* | -0.02 | *0.89* | -0.04 | *0.77* |
|  | (RR) Final acc. % of undershoot |  | 52 |  | -0.09 | *0.52* | 0.01 | *0.92* | 0.13 | *0.37* | -0.12 | *0.41* | -0.10 | *0.48* | 0.06 | *0.69* | 0.11 | *0.44* |
|  | Area under fit (AUF) mean |  | 52 |  | 0.00 | *0.99* | 0.02 | *0.90* | -0.22 | *0.11* | -0.20 | *0.16* | 0.24 | *0.08* | -0.06 | *0.68* | -0.01 | *0.95* |
| **SV** | Latency grand mean |  | 52 |  | -0.15 | *0.27* | 0.07 | *0.63* | -0.02 | *0.87* | 0.10 | *0.49* | -0.03 | *0.85* | 0.08 | *0.59* | -0.04 | *0.77* |
|  | Accuracy grand mean |  | 52 |  | 0.12 | *0.41* | 0.04 | *0.77* | 0.00 | *0.98* | 0.03 | *0.85* | 0.21 | *0.13* | -0.09 | *0.54* | 0.09 | *0.53* |
|  | (RU) Accuracy % of undershoot |  | 52 |  | 0.04 | *0.77* | 0.12 | *0.38* | 0.15 | *0.28* | -0.14 | *0.31* | -0.16 | *0.27* | 0.18 | *0.20* | 0.14 | *0.31* |
|  | Final accuracy grand mean |  | 52 |  | 0.06 | *0.68* | 0.02 | *0.89* | 0.21 | *0.14* | 0.10 | *0.46* | 0.25 | *0.07* | 0.12 | *0.39* | 0.25 | *0.07* |
|  | (RU) Final acc. % of undershoot |  | 52 |  | 0.17 | *0.22* | 0.05 | *0.70* | -0.02 | *0.86* | -0.18 | *0.21* | 0.00 | *1.00* | 0.14 | *0.33* | 0.07 | *0.62* |
|  | Area under fit (AUF) mean |  | 52 |  | 0.02 | *0.87* | 0.21 | *0.13* | -0.06 | *0.66* | -0.08 | *0.58* | 0.20 | *0.15* | -0.11 | *0.44* | -0.06 | *0.69* |
| **SPH, 0.1Hz** | Velocity gain rightward |  | 52 |  | -0.06 | *0.66* | -0.06 | *0.65* | 0.02 | *0.91* | 0.17 | *0.23* | 0.01 | *0.96* | -0.40 | ***0.00*** | -0.15 | *0.30* |
|  | Velocity gain leftward |  | 52 |  | 0.10 | *0.50* | -0.02 | *0.89* | 0.16 | *0.27* | 0.18 | *0.20* | 0.05 | *0.72* | -0.05 | *0.75* | 0.22 | *0.11* |
|  | Velocity saccade, % |  | 52 |  | 0.24 | *0.09* | 0.13 | *0.35* | 0.14 | *0.31* | 0.14 | *0.32* | -0.16 | *0.25* | 0.22 | *0.11* | 0.07 | *0.62* |
|  | Position gain |  | 52 |  | 0.01 | *0.92* | -0.11 | *0.43* | -0.09 | *0.51* | -0.14 | *0.34* | 0.13 | *0.37* | -0.39 | ***0.00*** | 0.09 | *0.53* |
|  | Initiation latency |  | 52 |  | 0.06 | *0.68* | -0.18 | *0.19* | -0.18 | *0.20* | -0.09 | *0.54* | 0.10 | *0.48* | 0.00 | *0.98* | 0.15 | *0.29* |
| **SPH, 0.75Hz** | Velocity gain rightward |  | 52 |  | 0.01 | *0.97* | -0.10 | *0.49* | -0.04 | *0.76* | 0.00 | *0.99* | 0.03 | *0.83* | 0.00 | *0.98* | -0.01 | *0.92* |
|  | Velocity gain leftward |  | 52 |  | 0.02 | *0.91* | 0.03 | *0.81* | -0.22 | *0.12* | -0.07 | *0.61* | 0.13 | *0.36* | -0.29 | ***0.04*** | -0.16 | *0.24* |
|  | Velocity saccade, % |  | 52 |  | -0.20 | *0.15* | 0.16 | *0.25* | 0.08 | *0.59* | 0.13 | *0.37* | 0.07 | *0.64* | 0.14 | *0.32* | 0.01 | *0.93* |
|  | Position gain |  | 52 |  | -0.16 | *0.27* | -0.15 | *0.30* | -0.07 | *0.60* | -0.06 | *0.66* | 0.03 | *0.81* | -0.16 | *0.26* | -0.20 | *0.15* |
|  | Initiation latency |  | 52 |  | -0.15 | *0.28* | 0.13 | *0.37* | -0.01 | *0.97* | 0.14 | *0.32* | -0.14 | *0.33* | 0.04 | *0.79* | -0.14 | *0.33* |
| **SPV, 0.1Hz** | Velocity gain up |  | 52 |  | 0.36 | ***0.01*** | -0.07 | *0.60* | 0.07 | *0.61* | -0.12 | *0.41* | 0.08 | *0.57* | 0.05 | *0.72* | 0.15 | *0.30* |
|  | Velocity gain down |  | 52 |  | -0.02 | *0.89* | -0.06 | *0.69* | 0.11 | *0.44* | 0.10 | *0.50* | 0.05 | *0.72* | 0.11 | *0.46* | 0.23 | *0.10* |
|  | Velocity saccade, % |  | 52 |  | -0.05 | *0.73* | 0.25 | *0.07* | 0.14 | *0.31* | 0.01 | *0.95* | -0.02 | *0.89* | 0.20 | *0.16* | 0.08 | *0.59* |
|  | Position gain |  | 52 |  | 0.20 | *0.17* | 0.01 | *0.94* | 0.01 | *0.96* | 0.04 | *0.77* | 0.02 | *0.88* | -0.10 | *0.47* | -0.03 | *0.84* |
|  | Initiation latency |  | 52 |  | 0.04 | *0.80* | 0.20 | *0.16* | -0.10 | *0.47* | 0.04 | *0.78* | 0.20 | *0.15* | 0.02 | *0.91* | -0.20 | *0.17* |
| **SPV, 0.75Hz** | Velocity gain up |  | 52 |  | 0.06 | *0.68* | -0.15 | *0.27* | -0.11 | *0.45* | -0.04 | *0.80* | -0.02 | *0.90* | -0.16 | *0.25* | -0.15 | *0.28* |
|  | Velocity gain down |  | 52 |  | 0.01 | *0.97* | 0.02 | *0.87* | -0.05 | *0.71* | -0.14 | *0.31* | -0.14 | *0.31* | 0.01 | *0.92* | 0.12 | *0.39* |
|  | Velocity saccade, % |  | 52 |  | 0.05 | *0.74* | 0.07 | *0.61* | 0.15 | *0.30* | 0.05 | *0.73* | -0.06 | *0.68* | 0.04 | *0.78* | -0.12 | *0.40* |
|  | Position gain |  | 52 |  | 0.15 | *0.30* | -0.06 | *0.69* | -0.03 | *0.84* | -0.20 | *0.16* | 0.01 | *0.97* | -0.27 | ***0.05*** | -0.18 | *0.21* |
|  | Initiation latency |  | 52 |  | -0.05 | *0.73* | -0.17 | *0.22* | -0.11 | *0.42* | 0.15 | *0.29* | -0.01 | *0.96* | -0.16 | *0.26* | -0.14 | *0.32* |
| **OKN, 20°/sec** | Average gain |  | 50 |  | 0.09 | *0.54* | -0.23 | *0.11* | -0.14 | *0.33* | 0.21 | *0.15* | 0.27 | *0.06* | -0.15 | *0.31* | -0.16 | *0.27* |
|  | Area under fit (AUF) mean |  | 50 |  | -0.14 | *0.34* | -0.09 | *0.54* | -0.28 | ***0.05*** | 0.03 | *0.85* | 0.08 | *0.57* | -0.10 | *0.47* | -0.25 | *0.07* |
| **OKN, 60°/sec** | Average gain |  | 49 |  | 0.07 | *0.65* | -0.16 | *0.26* | -0.12 | *0.42* | 0.14 | *0.34* | 0.36 | ***0.01*** | -0.12 | *0.42* | -0.05 | *0.72* |
|  | Area under fit (AUF) mean |  | 49 |  | -0.18 | *0.22* | 0.00 | *0.98* | -0.38 | ***0.01*** | -0.01 | *0.92* | 0.21 | *0.16* | -0.10 | *0.48* | -0.07 | *0.65* |
| **SVV** | Overall error mean |  | 50 |  | 0.16 | *0.26* | 0.24 | *0.10* | -0.08 | *0.59* | -0.32 | ***0.02*** | 0.28 | ***0.05*** | 0.03 | *0.81* | 0.16 | *0.28* |
| **ART** | Mean latency |  | 51 |  | 0.12 | *0.40* | 0.13 | *0.35* | 0.21 | *0.13* | -0.01 | *0.97* | -0.08 | *0.58* | -0.04 | *0.76* | 0.15 | *0.31* |
| **VRT** | Mean latency |  | 50 |  | -0.06 | *0.67* | 0.21 | *0.14* | 0.17 | *0.23* | -0.22 | *0.13* | -0.02 | *0.91* | -0.20 | *0.16* | 0.03 | *0.82* |
| **SRT** | *Saccadic metrics:* |  |  |  |  |  |  |  |  |  |  |  |  |  |  |  |  |  |
|  | Latency grand mean |  | 52 |  | -0.08 | *0.56* | 0.14 | *0.31* | 0.03 | *0.82* | 0.10 | *0.50* | -0.14 | *0.33* | 0.03 | *0.84* | -0.26 | *0.06* |
|  | Latency mean, rightward |  | 52 |  | -0.08 | *0.56* | 0.15 | *0.30* | 0.03 | *0.84* | 0.00 | *0.97* | -0.13 | *0.34* | 0.06 | *0.70* | -0.20 | *0.17* |
|  | Latency mean, leftward |  | 52 |  | -0.09 | *0.51* | 0.11 | *0.45* | 0.00 | *0.98* | 0.19 | *0.18* | -0.12 | *0.40* | -0.04 | *0.77* | -0.28 | ***0.05*** |
|  | (LR) Acc. % of undershoot |  | 32 |  | -0.05 | *0.80* | -0.05 | *0.79* | -0.12 | *0.50* | -0.12 | *0.50* | 0.19 | *0.29* | 0.06 | *0.75* | 0.21 | *0.25* |
|  | (RR) Acc. % of undershoot |  | 32 |  | -0.17 | *0.34* | -0.07 | *0.69* | -0.12 | *0.51* | -0.30 | *0.09* | -0.10 | *0.58* | -0.25 | *0.17* | 0.23 | *0.20* |
|  | Final acc. grand mean |  | 52 |  | 0.12 | *0.40* | -0.19 | *0.18* | -0.07 | *0.63* | 0.17 | *0.22* | 0.01 | *0.96* | 0.10 | *0.47* | -0.01 | *0.93* |
|  | *Motor metrics:* |  |  |  |  |  |  |  |  |  |  |  |  |  |  |  |  |  |
|  | Latency mean, right button |  | 50 |  | 0.12 | *0.41* | 0.20 | *0.16* | 0.11 | *0.46* | -0.16 | *0.25* | 0.21 | *0.14* | -0.14 | *0.34* | 0.25 | *0.08* |
|  | Latency mean, left button |  | 50 |  | 0.07 | *0.62* | 0.19 | *0.19* | 0.16 | *0.28* | -0.17 | *0.24* | 0.14 | *0.33* | -0.14 | *0.34* | 0.15 | *0.30* |
| **PS** | (L) % of predicted |  | 52 |  | 0.07 | *0.61* | 0.10 | *0.46* | 0.05 | *0.71* | 0.25 | *0.07* | 0.10 | *0.49* | 0.05 | *0.72* | -0.10 | *0.49* |
|  | (R) % of predicted |  | 52 |  | 0.16 | *0.25* | 0.12 | *0.40* | 0.08 | *0.58* | 0.29 | ***0.04*** | 0.13 | *0.36* | -0.08 | *0.55* | -0.15 | *0.27* |
|  |  |  |  |  |  |  |  |  |  |  |  |  |  |  |  |  |  |  |

**Table S.6. Correlation Between the Comorbidities (Reported at Time of Testing) and OVRT-C Tests**

| **Tests** | **Metrics** |  | **N** |  | **Anxiety** | | **Depression** | | **Insomnia** | | **Motion sickness** | |
| --- | --- | --- | --- | --- | --- | --- | --- | --- | --- | --- | --- | --- |
|  |  |  |  |  | **Rho** | ***p*** | **Rho** | ***p*** | **Rho** | ***p*** | **Rho** | ***p*** |
|  |  |  |  |  |  |  |  |  |  |  |  |  |
| **SH** | Latency grand mean |  | 52 |  | -0.12 | *0.38* | -0.05 | *0.71* | 0.06 | *0.66* | -0.15 | *0.28* |
|  | Accuracy grand mean |  | 52 |  | -0.17 | *0.22* | 0.03 | *0.83* | 0.07 | *0.61* | 0.28 | ***0.04*** |
|  | (RR) Accuracy % of undershoot |  | 52 |  | 0.05 | *0.73* | -0.19 | *0.17* | -0.12 | *0.40* | -0.06 | *0.68* |
|  | Final accuracy grand mean |  | 52 |  | -0.17 | *0.24* | -0.06 | *0.69* | -0.04 | *0.78* | 0.26 | *0.06* |
|  | (RR) Final acc. % of undershoot |  | 52 |  | 0.23 | *0.09* | -0.05 | *0.73* | -0.10 | *0.48* | -0.06 | *0.69* |
|  | Area under fit (AUF) mean |  | 52 |  | -0.04 | *0.80* | 0.23 | *0.10* | 0.13 | *0.37* | 0.32 | ***0.02*** |
| **SV** | Latency grand mean |  | 52 |  | 0.04 | *0.79* | -0.02 | *0.88* | 0.09 | *0.52* | -0.14 | *0.31* |
|  | Accuracy grand mean |  | 52 |  | -0.30 | ***0.03*** | -0.18 | *0.20* | 0.17 | *0.22* | 0.04 | *0.79* |
|  | (RU) Accuracy % of undershoot |  | 52 |  | 0.18 | *0.20* | 0.11 | *0.42* | -0.06 | *0.69* | 0.19 | *0.19* |
|  | Final accuracy grand mean |  | 52 |  | -0.19 | *0.18* | -0.12 | *0.38* | -0.03 | *0.81* | 0.01 | *0.94* |
|  | (RU) Final acc. % of undershoot |  | 52 |  | 0.28 | ***0.04*** | -0.01 | *0.95* | 0.22 | *0.12* | 0.37 | ***0.01*** |
|  | Area under fit (AUF) mean |  | 52 |  | -0.15 | *0.30* | 0.06 | *0.65* | 0.15 | *0.29* | 0.04 | *0.78* |
| **SPH, 0.1Hz** | Velocity gain rightward |  | 52 |  | -0.19 | *0.19* | 0.05 | *0.71* | -0.08 | *0.59* | -0.12 | *0.39* |
|  | Velocity gain leftward |  | 52 |  | -0.26 | *0.06* | -0.05 | *0.71* | -0.06 | *0.65* | -0.08 | *0.56* |
|  | Velocity saccade, % |  | 52 |  | 0.20 | *0.15* | 0.13 | *0.36* | -0.01 | *0.95* | -0.06 | *0.65* |
|  | Position gain |  | 52 |  | -0.22 | *0.11* | -0.22 | *0.12* | -0.20 | *0.16* | -0.12 | *0.39* |
|  | Initiation latency |  | 52 |  | 0.05 | *0.74* | 0.07 | *0.61* | 0.13 | *0.37* | 0.06 | *0.65* |
| **SPH, 0.75Hz** | Velocity gain rightward |  | 52 |  | -0.24 | *0.09* | 0.04 | *0.77* | -0.02 | *0.89* | -0.01 | *0.95* |
|  | Velocity gain leftward |  | 52 |  | -0.25 | *0.08* | -0.02 | *0.88* | 0.05 | *0.74* | 0.08 | *0.59* |
|  | Velocity saccade, % |  | 52 |  | -0.05 | *0.70* | 0.02 | *0.90* | 0.11 | *0.42* | -0.10 | *0.47* |
|  | Position gain |  | 52 |  | -0.29 | ***0.03*** | -0.12 | *0.40* | -0.08 | *0.59* | -0.08 | *0.58* |
|  | Initiation latency |  | 52 |  | 0.02 | *0.89* | -0.04 | *0.76* | 0.05 | *0.72* | -0.21 | *0.13* |
| **SPV, 0.1Hz** | Velocity gain up |  | 52 |  | -0.10 | *0.50* | -0.15 | *0.30* | 0.25 | *0.08* | 0.34 | ***0.01*** |
|  | Velocity gain down |  | 52 |  | -0.22 | *0.11* | 0.05 | *0.71* | -0.04 | *0.76* | 0.07 | *0.62* |
|  | Velocity saccade, % |  | 52 |  | 0.18 | *0.20* | 0.08 | *0.56* | -0.07 | *0.60* | -0.26 | *0.06* |
|  | Position gain |  | 52 |  | -0.20 | *0.15* | -0.09 | *0.51* | -0.07 | *0.64* | 0.08 | *0.56* |
|  | Initiation latency |  | 52 |  | 0.28 | ***0.04*** | 0.28 | ***0.04*** | 0.20 | *0.16* | 0.18 | *0.21* |
| **SPV, 0.75Hz** | Velocity gain up |  | 52 |  | -0.19 | *0.17* | -0.18 | *0.20* | 0.26 | *0.06* | 0.37 | ***0.01*** |
|  | Velocity gain down |  | 52 |  | -0.10 | *0.50* | 0.09 | *0.51* | 0.00 | *0.99* | 0.21 | *0.14* |
|  | Velocity saccade, % |  | 52 |  | -0.05 | *0.70* | -0.13 | *0.36* | 0.03 | *0.85* | -0.12 | *0.41* |
|  | Position gain |  | 52 |  | -0.21 | *0.13* | -0.27 | *0.06* | 0.11 | *0.43* | 0.23 | *0.10* |
|  | Initiation latency |  | 52 |  | 0.29 | ***0.04*** | 0.08 | *0.58* | 0.00 | *0.99* | -0.09 | *0.52* |
| **OKN, 20°/sec** | Average gain |  | 50 |  | 0.07 | *0.64* | -0.10 | *0.47* | -0.07 | *0.63* | 0.18 | *0.20* |
|  | Area under fit (AUF) mean |  | 50 |  | -0.22 | *0.12* | 0.11 | *0.44* | 0.08 | *0.60* | 0.17 | *0.25* |
| **OKN, 60°/sec** | Average gain |  | 49 |  | 0.14 | *0.33* | -0.13 | *0.38* | -0.11 | *0.46* | 0.09 | *0.54* |
|  | Area under fit (AUF) mean |  | 49 |  | -0.03 | *0.84* | 0.26 | *0.07* | 0.06 | *0.71* | 0.00 | *0.98* |
| **SVV** | Overall error mean |  | 50 |  | -0.05 | *0.73* | 0.07 | *0.65* | 0.04 | *0.79* | 0.05 | *0.71* |
| **ART** | Mean latency |  | 51 |  | 0.07 | *0.63* | 0.19 | *0.19* | 0.06 | *0.69* | 0.14 | *0.34* |
| **VRT** | Mean latency |  | 50 |  | 0.09 | *0.55* | 0.14 | *0.33* | -0.08 | *0.57* | -0.04 | *0.80* |
| **SRT** | *Saccadic metrics:* |  |  |  |  |  |  |  |  |  |  |  |
|  | Latency grand mean |  | 52 |  | -0.04 | *0.78* | 0.23 | *0.10* | 0.14 | *0.33* | -0.15 | *0.28* |
|  | Latency mean, rightward |  | 52 |  | -0.02 | *0.88* | 0.21 | *0.14* | 0.10 | *0.47* | -0.20 | *0.16* |
|  | Latency mean, leftward |  | 52 |  | -0.15 | *0.30* | 0.18 | *0.20* | 0.09 | *0.52* | -0.15 | *0.27* |
|  | (LR) Acc. % of undershoot |  | 32 |  | 0.18 | *0.33* | -0.11 | *0.54* | 0.12 | *0.51* | 0.01 | *0.98* |
|  | (RR) Acc. % of undershoot |  | 32 |  | -0.11 | *0.54* | -0.01 | *0.95* | 0.07 | *0.71* | 0.20 | *0.27* |
|  | Final acc. grand mean |  | 52 |  | 0.08 | *0.58* | 0.21 | *0.13* | -0.19 | *0.17* | -0.08 | *0.58* |
|  | *Motor metrics:* |  |  |  |  |  |  |  |  |  |  |  |
|  | Latency mean, right button |  | 50 |  | 0.09 | *0.54* | -0.07 | *0.65* | 0.13 | *0.37* | 0.04 | *0.79* |
|  | Latency mean, left button |  | 50 |  | 0.11 | *0.46* | -0.02 | *0.89* | 0.13 | *0.37* | 0.01 | *0.93* |
| **PS** | (L) % of predicted |  | 52 |  | -0.06 | *0.65* | 0.13 | *0.37* | 0.01 | *0.97* | -0.06 | *0.69* |
|  | (R) % of predicted |  | 52 |  | -0.21 | *0.13* | 0.12 | *0.40* | -0.06 | *0.69* | -0.11 | *0.43* |
|  |  |  |  |  |  |  |  |  |  |  |  |  |

**Table S.7.** **Correlation Between the Dizziness Handicap Inventory (DHI) Domains and OVRT-C Tests**

| **Tests** | **Metrics** |  | **N** |  | **Physical** | | **Emotional** | | **Functional** | | **Total** | | **Category** | |
| --- | --- | --- | --- | --- | --- | --- | --- | --- | --- | --- | --- | --- | --- | --- |
|  |  |  |  |  | **Rho** | ***p*** | **Rho** | ***p*** | **Rho** | ***p*** | **Rho** | ***p*** | **Rho** | ***p*** |
|  |  |  |  |  |  |  |  |  |  |  |  |  |  |  |
| **SH** | Latency grand mean |  | 51 |  | 0.06 | *0.67* | -0.10 | *0.49* | 0.20 | *0.16* | 0.10 | *0.46* | 0.04 | *0.79* |
|  | Accuracy grand mean |  | 51 |  | -0.10 | *0.48* | -0.12 | *0.38* | -0.34 | ***0.02*** | -0.22 | *0.13* | -0.26 | *0.07* |
|  | (RR) Accuracy % of undershoot |  | 51 |  | 0.20 | *0.17* | 0.07 | *0.63* | 0.34 | ***0.01*** | 0.27 | *0.06* | 0.27 | *0.06* |
|  | Final accuracy grand mean |  | 51 |  | -0.24 | *0.09* | -0.18 | *0.20* | -0.38 | ***0.01*** | -0.29 | ***0.04*** | -0.34 | ***0.01*** |
|  | (RR) Final acc. % of undershoot |  | 51 |  | 0.10 | *0.48* | -0.04 | *0.76* | 0.21 | *0.15* | 0.12 | *0.42* | 0.15 | *0.28* |
|  | Area under fit (AUF) mean |  | 51 |  | 0.19 | *0.17* | 0.25 | *0.07* | 0.04 | *0.77* | 0.16 | *0.26* | 0.16 | *0.28* |
| **SV** | Latency grand mean |  | 51 |  | 0.23 | *0.11* | -0.02 | *0.90* | 0.30 | ***0.03*** | 0.23 | *0.10* | 0.15 | *0.30* |
|  | Accuracy grand mean |  | 51 |  | -0.14 | *0.32* | -0.04 | *0.79* | -0.08 | *0.57* | -0.11 | *0.44* | -0.06 | *0.68* |
|  | (RU) Accuracy % of undershoot |  | 51 |  | 0.26 | *0.07* | 0.16 | *0.26* | 0.03 | *0.84* | 0.15 | *0.30* | 0.10 | *0.46* |
|  | Final accuracy grand mean |  | 51 |  | -0.01 | *0.92* | 0.13 | *0.35* | 0.06 | *0.67* | 0.06 | *0.67* | 0.10 | *0.49* |
|  | (RU) Final acc. % of undershoot |  | 51 |  | 0.15 | *0.29* | -0.07 | *0.63* | -0.18 | *0.20* | -0.06 | *0.66* | -0.11 | *0.46* |
|  | Area under fit (AUF) mean |  | 51 |  | 0.13 | *0.35* | 0.15 | *0.30* | 0.05 | *0.74* | 0.10 | *0.50* | 0.13 | *0.36* |
| **SPH, 0.1Hz** | Velocity gain rightward |  | 51 |  | -0.11 | *0.44* | -0.04 | *0.77* | 0.00 | *0.98* | -0.09 | *0.53* | -0.08 | *0.56* |
|  | Velocity gain leftward |  | 51 |  | -0.08 | *0.58* | -0.11 | *0.45* | -0.10 | *0.48* | -0.13 | *0.35* | -0.11 | *0.43* |
|  | Velocity saccade, % |  | 51 |  | 0.30 | ***0.03*** | 0.23 | ***0.10*** | 0.39 | ***0.01*** | 0.37 | ***0.01*** | 0.38 | ***0.01*** |
|  | Position gain |  | 51 |  | -0.32 | ***0.02*** | -0.13 | *0.37* | -0.23 | *0.10* | -0.25 | *0.07* | -0.26 | *0.06* |
|  | Initiation latency |  | 51 |  | -0.01 | *0.96* | 0.11 | *0.46* | 0.08 | *0.58* | 0.04 | *0.78* | 0.03 | *0.82* |
| **SPH, 0.75Hz** | Velocity gain rightward |  | 51 |  | -0.02 | *0.87* | 0.18 | *0.19* | -0.01 | *0.94* | 0.06 | *0.66* | 0.02 | *0.87* |
|  | Velocity gain leftward |  | 51 |  | -0.02 | *0.91* | 0.06 | *0.67* | -0.10 | *0.50* | -0.02 | *0.88* | -0.07 | *0.62* |
|  | Velocity saccade, % |  | 51 |  | 0.06 | *0.65* | -0.21 | *0.14* | -0.02 | *0.88* | -0.05 | *0.74* | -0.08 | *0.59* |
|  | Position gain |  | 51 |  | -0.20 | *0.15* | 0.04 | *0.80* | -0.18 | *0.21* | -0.12 | *0.42* | -0.16 | *0.25* |
|  | Initiation latency |  | 51 |  | 0.33 | ***0.02*** | -0.07 | *0.61* | 0.19 | *0.19* | 0.18 | *0.20* | 0.15 | *0.30* |
| **SPV, 0.1Hz** | Velocity gain up |  | 51 |  | 0.14 | *0.31* | -0.04 | *0.78* | -0.05 | *0.71* | 0.02 | *0.88* | -0.03 | *0.85* |
|  | Velocity gain down |  | 51 |  | 0.16 | *0.26* | -0.07 | *0.65* | 0.12 | *0.41* | 0.09 | *0.54* | 0.12 | *0.39* |
|  | Velocity saccade, % |  | 51 |  | 0.22 | *0.13* | 0.12 | *0.41* | 0.17 | *0.24* | 0.18 | *0.21* | 0.18 | *0.20* |
|  | Position gain |  | 51 |  | 0.16 | *0.25* | 0.21 | *0.13* | 0.26 | *0.07* | 0.26 | *0.07* | 0.27 | ***0.05*** |
|  | Initiation latency |  | 51 |  | 0.09 | *0.55* | 0.20 | *0.17* | 0.05 | *0.74* | 0.09 | *0.52* | 0.13 | *0.36* |
| **SPV, 0.75Hz** | Velocity gain up |  | 51 |  | 0.09 | *0.55* | 0.09 | *0.52* | 0.08 | *0.57* | 0.12 | *0.40* | 0.05 | *0.71* |
|  | Velocity gain down |  | 51 |  | 0.10 | *0.48* | 0.17 | *0.24* | 0.04 | *0.80* | 0.11 | *0.45* | 0.13 | *0.35* |
|  | Velocity saccade, % |  | 51 |  | -0.10 | *0.50* | -0.15 | *0.29* | -0.01 | *0.95* | -0.08 | *0.55* | -0.07 | *0.62* |
|  | Position gain |  | 51 |  | -0.10 | *0.48* | 0.13 | *0.37* | 0.02 | *0.89* | 0.03 | *0.82* | -0.02 | *0.88* |
|  | Initiation latency |  | 51 |  | 0.03 | *0.84* | -0.01 | *0.97* | 0.16 | *0.27* | 0.08 | *0.58* | 0.02 | *0.92* |
| **OKN, 20°/sec** | Average gain |  | 49 |  | -0.17 | *0.23* | -0.04 | *0.76* | -0.09 | *0.54* | -0.11 | *0.44* | -0.12 | *0.41* |
|  | Area under fit (AUF) mean |  | 49 |  | 0.17 | *0.24* | 0.26 | *0.07* | 0.17 | *0.23* | 0.23 | *0.11* | 0.23 | *0.12* |
| **OKN, 60°/sec** | Average gain |  | 48 |  | -0.08 | *0.59* | 0.06 | *0.67* | 0.00 | *0.99* | 0.00 | *0.98* | -0.02 | *0.91* |
|  | Area under fit (AUF) mean |  | 48 |  | 0.12 | *0.42* | 0.27 | *0.06* | 0.20 | *0.16* | 0.21 | *0.15* | 0.24 | *0.10* |
| **SVV** | Overall error mean |  | 49 |  | 0.13 | *0.37* | 0.19 | *0.19* | -0.08 | *0.61* | 0.08 | *0.61* | 0.04 | *0.77* |
| **ART** | Mean latency |  | 50 |  | 0.12 | *0.42* | 0.29 | ***0.04*** | 0.27 | *0.06* | 0.23 | *0.11* | 0.24 | *0.09* |
| **VRT** | Mean latency |  | 49 |  | 0.07 | *0.63* | 0.25 | *0.08* | 0.17 | *0.23* | 0.19 | *0.19* | 0.17 | *0.26* |
| **SRT** | *Saccadic metrics:* |  |  |  |  |  |  |  |  |  |  |  |  |  |
|  | Latency grand mean |  | 51 |  | 0.06 | *0.69* | -0.24 | *0.10* | -0.01 | *0.94* | -0.05 | *0.75* | -0.05 | *0.70* |
|  | Latency mean, rightward |  | 51 |  | 0.01 | *0.96* | -0.31 | ***0.03*** | -0.11 | *0.46* | -0.12 | *0.39* | -0.11 | *0.42* |
|  | Latency mean, leftward |  | 51 |  | 0.07 | *0.62* | -0.18 | *0.20* | 0.05 | *0.73* | 0.00 | *1.00* | -0.02 | *0.89* |
|  | (LR) Acc. % of undershoot |  | 31 |  | -0.03 | *0.86* | -0.23 | *0.20* | -0.04 | *0.82* | -0.10 | *0.61* | -0.12 | *0.51* |
|  | (RR) Acc. % of undershoot |  | 31 |  | 0.08 | *0.66* | -0.29 | *0.12* | -0.04 | *0.82* | -0.06 | *0.73* | -0.06 | *0.73* |
|  | Final acc. grand mean |  | 51 |  | 0.06 | *0.70* | 0.14 | *0.34* | 0.13 | *0.38* | 0.13 | *0.35* | 0.07 | *0.63* |
|  | *Motor metrics:* |  |  |  |  |  |  |  |  |  |  |  |  |  |
|  | Latency mean, right button |  | 49 |  | 0.14 | *0.34* | 0.06 | *0.71* | 0.19 | *0.19* | 0.16 | *0.28* | 0.15 | *0.29* |
|  | Latency mean, left button |  | 49 |  | 0.21 | *0.15* | 0.19 | *0.19* | 0.29 | ***0.04*** | 0.27 | *0.06* | 0.26 | *0.07* |
| **PS** | (L) % of predicted |  | 51 |  | -0.01 | *0.94* | -0.02 | *0.91* | 0.02 | *0.92* | -0.01 | *0.93* | 0.06 | *0.69* |
|  | (R) % of predicted |  | 51 |  | 0.00 | *0.98* | 0.05 | *0.74* | 0.04 | *0.81* | 0.02 | *0.89* | 0.08 | *0.59* |
|  |  |  |  |  |  |  |  |  |  |  |  |  |  |  |
